# Identification of BET inhibitors (BETi) against solitary fibrous tumor (SFT) through high-throughput screening (HTS)

**DOI:** 10.1016/j.neo.2025.101244

**Published:** 2025-10-29

**Authors:** Jose L. Mondaza-Hernandez, David S. Moura, Yi Li, Jesus L. Marti, Paulino Gomez-Puertas, John T. Nguyen, Shuguang Wei, Bruce A. Posner, Clark A. Meyer, Leonidas Bleris, Javier Martin-Broto, Heather N. Hayenga

**Affiliations:** a Health Research Institute Fundacion Jimenez Diaz, Universidad Autonoma de Madrid (IIS/FJD-UAM), Madrid 28049, Spain; b University Hospital General de Villalba, Madrid 28400, Spain; c Department of Bioengineering, University of Texas at Dallas, Richardson, TX, 75080, USA; d Center for Systems Biology, University of Texas at Dallas, Richardson, TX, 75080, USA; e Molecular Modeling Group, Centro de Biologia Molecular Severo Ochoa (CBM, CSIC-UAM), Madrid 28049, Spain; f Department of Biochemistry, University of Texas Southwestern Medical Center, Dallas, TX, 75390, USA; g Department of Biological Sciences, University of Texas at Dallas, Richardson, TX, 75080, USA; h Medical Oncology Department, University Hospital Fundacion Jimenez Diaz, Madrid 28040, Spain

**Keywords:** BET inhibitor (BETi), Mivebresib, Solitary fibrous tumor (SFT), High-throughput screen (HTS)

## Abstract

Cancers, especially fusion oncoprotein (FO)-driven hematological cancers and sarcomas, often develop from a low number of key mutations. Solitary Fibrous Tumor (SFT) is a rare mesenchymal tumor driven by the NAB2-STAT6 oncofusion gene. Currently, the treatment options for SFT remain limited, with anti-angiogenic drugs providing only partial responses with an average survival of two years. We constructed SFT cell models harboring specific NAB2-STAT6 fusion transcripts using the CRISPR (Clustered Regularly Interspaced Short Palindromic Repeats) technology, and we used these cells as models of SFT. High-throughput drug screens demonstrated that the BET inhibitor Mivebresib can differentially reduce proliferation in SFT cell models. Subsequently, BET inhibitors Mivebresib and BMS-986158 efficiently reduced tumor growth in an SFT patient-derived xenograft (PDX) animal model. Furthermore, our data showed that NAB2-STAT6 fusions may lead to high levels of DNA damage in SFTs. Consequently, combining BET inhibitors with PARP (Poly (ADP-ribose) polymerase) inhibitors or with ATR inhibitors significantly enhanced anti-proliferative effects in SFT cells. Taken together, this study establishes BET inhibitors Mivebresib and BMS-986158 as promising anti-SFT agents.

## Introduction

Solitary fibrous tumor (SFT) is a rare mesenchymal tumor that demonstrates fibroblastic differentiation and may arise anywhere in the body. In 2013, it was discovered that nearly all solitary fibrous tumors have a version of a hallmark intrachromosomal fusion gene between NAB2 and STAT6 on chromosome 12 [1,2]. Since then, at least 6 distinct fusion types that account for the observed pathologic variation and tumor aggressiveness have been identified in SFTs, NAB2*_exon6_::*STAT6*_exon16/17_* and NAB2*_exon4_::*STAT6*_exon2_* being the most frequent variants [3]. Previous studies suggest that NAB2-STAT6 is the oncogenic driver; otherwise, the TMB in SFT patients has as few as 0 mutations/Mb^1,3,4^. Yet even though this single fusion gene drives tumorigenicity, therapeutic options and, most importantly, targeted studies and clinical trials are lacking. Surgery and/or radiation are the first line of treatment against this tumor; however, for many, this becomes challenging as cancer can travel to inoperable areas or recur in locations already irradiated. Anti-angiogenic drugs developed to treat other cancers, including kidney, ovarian, colorectal, lung, and brain, are the best therapeutic options for advanced SFT [[5], [6], [7], [8]]. However, none of the currently available systemic therapies enable complete remission, with the best response being a partial response or stable disease for several months. The average survival rate of patients on the chemotherapies available is 2 years [9]. One of the major bottlenecks in the SFT research field is the lack of *in vitro* and *in vivo* disease models.

BET (bromodomain and extraterminal) proteins are epigenetic readers that recognize acetylated lysine residues on histones and nonhistone proteins, functioning mainly as scaffolds that recruit chromatin regulators to active gene promoters and enhancers [10]. Through their bromodomains (BD) and extraterminal (ET) domains, they facilitate transcription elongation, enhancer-promoter communication, chromatin remodeling, and RNA polymerase II pause release [[11], [12], [13], [14]]. In addition, BET proteins play crucial roles in maintaining genome integrity by coordinating the repair of DNA double-strand breaks (DSBs), replication initiation, and transcription-replication dynamics [[15], [16], [17]]. BET inhibitors (BETi), such as JQ1, have demonstrated antitumor activity in several fusion-driven sarcomas, including rhabdomyosarcoma (RMS) and Ewing sarcoma (ES), by disrupting BRD4-dependent transcriptional activity, reducing oncogene expression (e.g., MYC), and impairing super-enhancer function [[18], [19], [20], [21]].

In this study, we used CRISPR (Clustered Regularly Interspaced Short Palindromic Repeats) based genome editing to engineer SFT cell models with specific NAB2-STAT6 gene fusions [22,23], which were subsequently applied to a high-throughput screening (HTS) platform with primary and secondary studies. We identified compounds that selectively disrupt the oncogenic NAB2-STAT6 fusion-driven signaling, which could later be used as systemic therapeutic agents for SFT. The anti-tumor efficacy of a particular compound, BET (bromodomain and extraterminal proteins) inhibitor Mivebresib, was validated using an SFT patient-derived xenograft (PDX) mouse model.

## Materials and methods

### Mammalian cell culture

The HCT116 cells were acquired from the American Type Culture Collection (ATCC, catalog number: CCL-247) and maintained at 37 °C, 100 % humidity, and 5 % CO_2_. The cells were grown in Dulbecco’s modified Eagle’s medium (DMEM media, Invitrogen, catalog number: 11965–1181) supplemented with 10 % fetal bovine serum (FBS, Invitrogen, catalog number: 26140), 0.1 mM MEM non-essential amino acids (Invitrogen, catalog number: 11140–050), and 100 units/mL of Penicillin and 100 units/mL of Streptomycin (Penicillin-Streptomycin liquid, Invitrogen, catalog number: 15140). To pass the cells, the adherent culture was first washed with PBS (Dulbecco’s Phosphate Buffered Saline, Mediatech, catalog number: 21-030-CM), then trypsinized with Trypsin-EDTA (0.25 % Trypsin with EDTA, Invitrogen, catalog number: 25200), and finally diluted in fresh medium. The same protocol was used for maintaining NS-poly cells, except that hygromycin (200 µg/mL, Thermo Fisher Scientific, catalog number: 10687010) was included in the complete growth medium for NS-poly cells.

The hTERT-immortalized human lung fibroblast cell line (Lf) was acquired from the American Type Culture Collection (catalog number: CRL-4058) and maintained at 37 °C, 100 % humidity, and 5 % CO_2_. The cells were grown in Fibroblast Basal Medium (ATCC, catalog Number: PCS-201-030) supplemented with Fibroblast Growth Kit-Low serum (ATCC, catalog number: PCS-201-041), and 0.3 µg/mL of puromycin (Gibco, catalog number: A1113803). To pass the cells, the adherent culture was first washed with PBS, then trypsinized with Trypsin-EDTA for Primary Cells (ATCC, Catalog number: PCS-999-003 at 37 °C for 10 min, and finally diluted in fresh medium.

The primary SFT cell line (Moffitt-ns) was harvested and isolated from Moffitt Cancer Center (MCC). Under approval by the Total Cancer Care (TCC) program at Moffitt, SFT tissue samples were resected [23]. The INT-SFT cell was gifted from Dr. Roberta Maestro’s group at the Oncology Referral Center (Centro di Riferimento Oncologico) [23]. The SKUT-1 cells were acquired from Dr. Javier Martin-Broto’s group at Advanced Therapies and Biomarkers in Sarcomas (ATBSarc). The IEC139 and CP0024 cell lines were established from female SFT and leiomyosarcoma patients, respectively, in the laboratory of Dr. Javier Martin-Broto.

Moffitt-ns, INT-SFT, IEC139, and CP0024 cells were maintained in RPMI-1640 media (Thermo Fisher Scientific, catalog number: 11-875-085) containing 10 % fetal bovine serum (FBS), MEM non-essential amino acids, and penicillin/streptomycin. SKUT-1 cells were maintained in DMEM media containing 10 % fetal bovine serum (FBS), MEM non-essential amino acids, and penicillin/streptomycin.

### High-throughput screening (HTS)

For High-Throughput Screening (HTS), a live-cell, high-content assay was employed to measure the fraction of dead/dying cells as a function of time. Optimized number of cells (400 cells/well for NS-poly, 600 cells/well for Lf, and 900 cells/well for Moffitt-ns) were plated into 384-well plates (Greiner Bio-one, catalog number: 781091) in the complete growth medium containing two dyes: CellTracker Deep Red (1:5,000, Thermo Fisher Scientific, catalog number: C34565) and DRAQ7 (1:200, Abcam, catalog number: ab109202). The cells were incubated at 37 °C, 5 % CO2 incubator overnight before chemical treatment using an acoustic ejection dispensing system (Echo 655 Liquid Handler, Beckman, Inc, catalog number: 001-16080). Next, images were taken for the CellTracker Red CMTPX (Ex: 561 nm) and DRAQ7 (Ex: 647 nm) channels at 0, 24, 48, and 72 h with a 20x/0.45 air objective using an In Cell Analyzer 6000 Cell Imaging System (GE Healthcare). Four fields of view were captured per well.

Three metrics were used in data analysis: 1) AUC effects: the area-under-the-curve (AUC) of the drug response results over all time points; 2) FinalTimepoint effects: the drug response results at the final timepoint (72 h); 3) CTG effects: the cell viability assay (Promega, CellTiter-Glo Luminescent Cell Viability assay) results at the final timepoint (72 h).

### CellTiter-Glo luminescent cell viability assay

The CellTiter-Glo Luminescent Cell Viability kit was purchased from Promega (catalog number: G7573). The cell viability assays were performed according to the manufacturer's recommendations. Briefly, the CellTiter-Glo reagent was added to each well in a 384-well plate (10 µL of a 1:2 dilution in PBS with 1 % Triton X-100) using the Biomek i7 Automated Workstation system (Beckman Coulter, Inc., catalog number: B87581). The plates were incubated for 10 min at room temperature on a shaker, and subsequently, the luminescence was measured using an EnVision multimode plate reader (Perkin-Elmer, catalog number: 2105-0010).

### MTS cell viability assay

All compounds were purchased from Selleck Chemicals. MTS assays were performed according to the manufacturer’s recommendations (Promega, CellTiter 96 Aqueous One Solution Cell Proliferation Assay, catalog number: G3582). Briefly, ∼5,000 cells were seeded in 96-well plates with 4 replicates. 16 h later, the drugs were added at different concentrations. 72 h later, MTS assays were performed by replacing the growth medium with a fresh medium containing 20 % MTS substrate, and absorbances were measured at 490 nm using a plate reader (Heales, catalog number: MB-580). For data analysis, GraphPad Prism 8 was used to calculate IC_50_ values.

### Flow cytometry-based apoptosis and cell cycle analysis

SFT cells were treated with candidate compounds or control DMSO. Next, for apoptosis analysis, the FITC Annexin V/propidium iodide (PI) Apoptosis Detection Kit (Immunostep) was used, following the manufacturer's protocol using a FACSCanto II or BD Accuri C6 Plus system (BD Biosciences). The FITC fluorescence channel was used to detect annexin V-positive cells (early apoptosis), and the PerCP channel was used to detect PI-positive cells (late apoptosis/necrosis). It should be noted that a sub-G1 population was identified in INT-SFT cells, which likely indicates an apoptotic subset with degraded DNA.

For cell cycle analysis, cells were treated with candidate compounds for 24 h and then fixed with 70 % ethanol for 1 h at 4 °C. After RNase A digestion, cells were stained with PI and analyzed for DNA content using the PE channel on the BD Accuri C6 Plus system. Data was then processed with BD FACS Diva and Floreada.io software.

### *In vivo* treatment with Mivebresib

Nude athymic mice (Jackson Laboratory) were implanted with 2-3 mm³ IEC139 PDX (patient-derived xenograft) tumor fragments. A minimum of 3 animals were necessary to detect a clinically relevant difference of 400mm [3] in tumor volume between the control-treated group and the Mivebresib-treated group. The test was performed with a power of 80 % and a statistical significance of 5 %. The standard deviation used in the test was 125mm [3]. Once tumors reached a volume of 150–200 mm³, the mice were randomly assigned to receive either vehicle or Mivebresib (Selleckchem). Mivebresib was prepared in 2 % DMSO and administered by oral gavage at a dose of 1 mg/kg body weight. The treatment followed an intermittent schedule, with dosing for 5 consecutive days followed by a 2-day break over 31 days or until tumor volume reached 1,500 mm³. Body weight and tumor volume were monitored every 2-3 days. Tumor measurements followed the same method as described in our previous report.

### Western blot (WB)

Cells were lysed using 1XRIPA buffer (1 M Tris–HCl pH 8, 0.5 M EDTA, Triton™ X-100, 10 % sodium deoxycholate, 10 % SDS, and 3 M NaCl), supplemented with protease and phosphatase inhibitors (all Sigma-Aldrich). Protein samples (20 μg) were separated by SDS-PAGE using a constant current of 90 V for stacking and 120 V for resolving acrylamide gels. Proteins were transferred to 0.2 μm pore-size Amersham nitrocellulose membranes (Cytiva) at 4 °C for 150 min at 200 mA constant current. Membranes were then blocked for 1 h with 5 % bovine serum albumin (BSA) or non-fat milk (PanReac AppliChem ITW Reagents) in 1X TBS 0.1 % Tween-20 (Bio-Rad). Primary antibodies were then applied overnight at 4 °C in BSA or milk, as recommended by the manufacturer. Next, after washing with 1X TBS-T, membranes were incubated with secondary antibodies: Rabbit Anti-Mouse IgG–Peroxidase (Sigma-Aldrich) or Goat Anti-Rabbit IgG H&L Peroxidase-conjugated (Abcam). Chemiluminescent detection was performed using ECL Prime (Cytiva), and images were acquired with a Chemidoc Imaging System (Bio-Rad). Band intensities were quantified using Image Lab software (Bio-Rad).

**Antibodies used in this study.**

**Table:** 

Target Protein	Reference	Supplier	Host species	Blocking agent	Dilution	Molecular weight (kDa)
**α-tubulin**	T9026	Sgma-Adrich	Mouse	Milk	0.74	50
**PARP-1**	51-6639GR	BDBiosciences	Mouse	BSA	0.56	116/89
**γ-H2AX (S-139)**	9718	Cell signaling	Rabbit	Milk	0.74	15
**p-ATR(S-428)**	720107	Thermo Fisher Scientific	Rabbit	Milk	0.56	300
**Cyclin D1**	ab16663	Abcam	Rabbit	Milk	0.56	36
**p21**	29475	Cell signaling	Rabbit	Milk	0.74	21
**Wee-1**	sc-9037	Santa Cruz Biotechnology	Rabbit	BSA	0.56	95
**RAD51**	PAS-27195	Invitrogen	Rabbit	Milk	0.74	37

### Real-time RT-PCR (Reverse Transcription-PCR)

For real-time RT-PCR assays, total RNAs were extracted using the RNeasy Mini Kit (Qiagen, catalog number: 74106). First-strand cDNAs were synthesized using a QuantiTect Reverse Transcription kit (500 ng RNA, Qiagen, catalog number: 205311). Next, quantitative PCR was performed using the KAPA SYBR FAST universal qPCR Kit (Kapa Biosystems, Wilmington, MA, USA, catalog number KK4601), with GAPDH as the internal control. The forward primer for GAPDH was 5′-AATCCCATCACCATCTTCCA-3′, and the reverse primer for GAPDH was 5′-TGGACTCCACGACGTACTCA-3′. The forward primer for NAB2-STAT6 was 5′-CGAAGCCACCTCTCGCAG-3′, and the reverse primer for NAB2-STAT6 was 5′-CTTGTAGTGGCTCCGGAAAG-3′. Quantitative analysis was performed using the 2−ΔΔCt method. Fold-change values were reported as means with standard deviations.

### Chou-Talalay and matrix combination analyses for synergy determination

IC50 concentrations were calculated for each candidate compound in SFT cell lines. Briefly, DMSO was used as the drug vehicle and negative control. In 96-well plates, 2 × 10^3^ cells were seeded and exposed to drug concentrations ranging from 10^−10^ to 10^-5^ M for Mivebresib and BMS-986158, and from 10^−9^ to 10^−4^ M for Rucaparib and Berzosertib, over 72 h. Drug combinations were prepared by pairing the lowest concentration of drug 1 with the lowest concentration of drug 2, followed by incrementally higher concentrations of drug 1 and drug 2, respectively.

The colorimetric MTS method (Promega) was used to measure cell viability, with absorbance recorded at 492 nm using a Heales MB-580 microplate absorbance reader (Shenzhen Huisong Technology Development). Cell viability values were normalized to the control condition. Subsequently, the non-linear model fit “log(inhibitor) vs. response – Variable slope (four parameters)” in Graphpad Prism 8.0 software (RRID: SCR_002798, San Diego, CA, USA) was used for data analysis. IC75 and IC90 values were determined in the same manner.

Combination index (CI) at different concentrations was then calculated as described by Chou-Talalay [24]:

$$CI=\;\frac{{\left(D\right)}_{1}}{{\left(D_{x}\right)}_{1}}+\;\frac{{\left(D\right)}_{2}}{{\left(D_{x}\right)}_{2}}$$

Where:

(D)_1_ and (D)_2_ are the concentrations of drug 1 or drug 2, respectively, in the combination that achieve a certain effect (IC50, IC75, IC90).

(D_x_)_1_ and (D_x_)_2_ are the concentrations as single agents of drug 1 or drug 2, respectively, that achieve the same effect.

The CI values are interpreted as:

Strong synergism: CI < 0.3

Synergism: CI between 0.3 and 0.7

Moderate to slight synergism: CI between 0.7 and 0.9

Nearly additive: CI between 0.9 and 1.1

Slight to moderate antagonism: CI between 1.1 and 1.45

Strong antagonism: CI > 1.45

For matrix combinations, BETi and DDRi were combined at concentrations ranging from 10⁻⁹ to 10⁻⁵ M in all possible pairwise combinations. The Combenefit software (RRID:SCR_027410) was used to evaluate drug synergy across the combination matrix using the Loewe additivity model, selected due to the potential overlap in mechanisms of action between the compounds. Highest single agent (HSA) method was also considered.

### RNA-seq analysis

INT-SFT and IEC139 cell lines were treated with 50 nM Mivebresib or BMS-986158 for 24 h. Total RNA was extracted as described above and subjected to RNA sequencing. Libraries were prepared and sequenced using Illumina NovaSeq X technology. RNA-seq reads were aligned to the human transcriptome using Salmon (RRID:SCR_017036) with GRCh38 as reference genome and Gencode (RRID:SCR_014966) as reference annotation. Gene-level counts were obtained from transcript-level estimates using the tximport package (RRID:SCR_016752) in R/Bioconductor. Differential expression was analyzed with DESeq2 (RRID:SCR_015687). Gene Set Enrichment Analysis (GSEA, RRID:SCR_003199) was performed using variance-stabilized counts and MSigDB (RRID:SCR_016863) gene sets (HALLMARK, KEGG, REACTOME, GO).

## Results

### Primary high-throughput screening (HTS) using the SFT NS-poly cells

Using the CRISPR/spCas9 system, we first generated an engineered SFT cell line (we named “NS-poly”) for the NAB2*_exon6_*::STAT6*_exon17_* fusion type by modifying a colorectal cancer cell line, HCT116. The HCT116 cell type was chosen due to its high transient transfection efficiency and the fact that no primary SFT cell lines were available in our lab at the time. Compared to prior engineered SFT cell models [4], the engineered NS-poly cells are heterozygous [22] and preserve all original NAB2-STAT6 gene fusion information, including endogenous NAB2 promoters, 5′-UTRs (5′-untranslated region) of NAB2, and 3′-UTRs of STAT6. Next, we performed a primary HTS assay using the NS-poly cells against the FDA-approved and experimental drug collection (∼2,600 compounds) [[25], [26], [27], [28], [29], [30], [31], [32]]. Briefly, NS-poly cells were seeded into 384-well assay plates and treated with the annotated chemical library at a final concentration of 5 µM with each compound. The assay plates were then processed as described in Materials and Methods/High-Throughput Screening (HTS). For quantitative analysis, the fractions of dead/dying cells (DRAQ7 cell count/CellTracker Deep Red cell count) were calculated for each well at all time points, which were used to plot the dose-response curves (e.g., **Supplementary Figure S1a** for Paclitaxel in NS-poly cells). Subsequently, we computed an area-under-the-curve (AUC) result for the treatment time course (e.g., **Supplementary Figure S1b** for Paclitaxel in NS-poly cells). We employed two normalization methods to evaluate the activity of library compounds. For the test population-based method, we calculated a robust mean and standard deviation for the test population (the library compound containing wells) and scaled compound activities to DMSO (arbitrarily set to 0 % effect). For the control-based method, we calculated the robust mean and standard deviation for the controls (vehicle control: DMSO alone and positive control: paclitaxel) and scaled compound activity (defined as observed minus vehicle mean) to the difference between these controls’ means.

For identification of compounds that can efficiently induce cell death in NS-poly cells, we utilized robust Z-scores for both (a) normalized activities at the final timepoint (72 h, named as Final Timepoint effects) and (b) normalized AUCs (named as AUC effects) [33]. Only compounds with robust Z-scores less than -3 were selected for the following secondary assays. 247 compounds were identified using the Final Timepoint effects (**Supplementary Table S1**), and 232 were determined using the AUC effects (**Supplementary Table S2**).

### Secondary high-throughput screening (HTS) using patient-derived Moffitt-ns and immortalized lung fibroblast cells

In our selection process for primary hits for secondary HTS assays, we first identified compounds that passed the primary screening criteria (robust Z-score < -3) and exerted suppression effects of >70 % for both the 72-hour endpoint (final timepoint effects) and the AUC measurements (AUC effects). In addition to the hits identified by the primary HTS, we included systemic agents currently used in or related to the treatment of SFT patients (e.g., the receptor tyrosine kinase inhibitor Sunitinib). In total, secondary screening included 104 compounds (**Supplementary Table S3**), comprising 93 from the primary screen and 11 from clinical applications. These compounds were obtained from either Selleck Chemicals (60) or the NIH (44).

Given its colorectal cancer background, our HCT116-based NS-poly cell model may additionally harbor oncogenic drivers other than the NAB2-STAT6 fusion (e.g., KRAS^G13D^ mutation [34]), which could muddle the interpretation of HTS results. Therefore, for the secondary high-throughput screening, we utilized a primary cell line derived from an SFT patient at Moffitt Cancer Center (named Moffitt-ns, fusion type: NAB2*_exon5_*::STAT6*_exon16_*) [23]. Finally, to match the fibrous background of Moffitt-ns, an hTERT-immortalized human lung fibroblast cell line (Lf) was used as the negative control. The same screening platform (dual dyes: CellTracker Deep Red and DRAQ7) from the primary screen was adopted for the secondary screen, except that: (a) Four doses (50 nM, 200 nM, 650 nM, and 2 µM for compounds from Selleck Chemicals; 125 nM, 500 nM, 1.6 µM, and 5 µM for compounds from the NIH Clinical Collection) were tested; and (b) an additional cell viability assay (Promega, CellTiter-Glo Luminescent Cell Viability assay) was performed at the final timepoint (CTG effects).

**Fig. 1:**
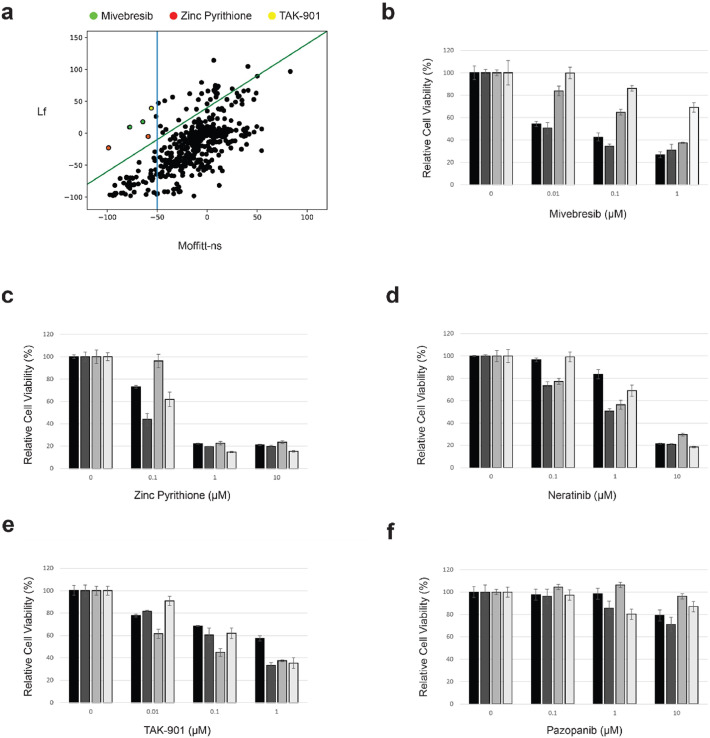
Identification of Mivebresib as an efficient and specific anti-SFT (Solitary Fibrous Tumor) agent. **(a)** Secondary high-throughput screening (HTS) was performed using the Moffitt-ns and immortalized lung fibroblast cells. The CTG effects of both cell lines under all four doses were plotted. The green line indicated the differences in suppression effects between Moffitt-ns and Lf > 40 %. The blue line indicated that the suppression effects at the highest dose should be > 50 % in Moffitt-ns cells. 3 compounds were shown (Mivebresib: green, Zinc Pyrithione: red, and TAK-901: yellow). **(b-f)** Confirmatory MTS assays were performed in two primary SFT cells (INT-SFT and IEC139) and two control LMS cells (SKUT-1 and CP0024). Only Mivebresib showed efficient and selective cell proliferation-suppressing effects in SFT cells. (): INT-SFT; (): IEC139; (): SKUT-1; (): CP0024.

To identify candidate compounds that can selectively, efficiently, and safely suppress the growth of Moffitt-ns cells, we applied three filtering conditions: 1) Selectivity: The differences in cell suppression between Moffitt-ns and control Lf are > 40 % for at least two doses; 2) Efficacy: Cell suppression at the highest dose should be > 50 % in Moffitt-ns cells; and, 3) Off-target toxicity: Cell suppression at the highest dose should be < 50 % in the control Lf cells. As shown in Fig. 1**a** and **Supplementary Tables S4** and **S5**, using these filtering conditions, we identified 3 candidates (Mivebresib, Zinc Pyrithione, and TAK-901) for the CTG effects, and 11 candidates (Mivebresib, Zinc Pyrithione, Staurosporine, Digoxin, Lanatoside C, Proscillaridin A, Disulfiram, Doxorubicin hydrochloride, Colchicine, Vinorelbine, and SW197775) for the final timepoint effects (**Supplementary Tables S6** and **S7**). Similarly, for the AUC effects (**Supplementary Tables S8** and **S9**), 8 hits were identified (Zinc Pyrithione, Staurosporine, Digoxin, Lanatoside C, Proscillaridin A, Doxorubicin hydrochloride, Colchicine, and SW197775).

### *In vitro* efficacy testing of Mivebresib using additional primary SFT cells

To determine candidate compounds for further efficacy studies, we focused on compounds that suppress the growth of SFT cancer cells. Thus, more weight was given to the CTG and final timepoint effects, and subsequently, two hits (Mivebresib, Zinc Pyrithione) were identified as present in both analyses (**Supplementary Tables S4-S7**). Notably, Zinc Pyrithione was also identified in the AUC effects analysis (**Supplementary Table S9**). For controls, we included three compounds from our secondary screening that did not pass the filtering conditions (Neratinib, TAK-901, and Pazopanib).

Our primary and secondary HTS assays were performed using specific NAB2-STAT6 fusion types (NAB2*_exon6_*::STAT6*_exon17_* for the primary screen and NAB2*_exon5_*::STAT6*_exon16_* for the secondary screen). To further test the robustness of our candidate compounds, after the completion of the primary and secondary HTS assays, we further procured two additional SFT patient-derived primary cell lines (INT-SFT with a fusion type: NAB2*_exon6_*::NAB2*_intron6_*::STAT6*_exon16_*; and IEC139 with a fusion type: NAB2*_exon6_*::STAT6*_exon16_*) and performed in vitro confirmatory efficacy testing (MTS Cell Viability assay) [23]. Additionally, to determine the specificity of our hits against SFTs, but not other soft tissue sarcomas, two Leiomyosarcoma cell lines (SKUT-1 and CP0024) were included. As shown in Fig. 1**b**, Mivebresib efficiently suppressed the cell growth in both SFT cell lines (IC_50_: 8.94 nM for INT-SFT, and 7.71 nM for IEC139), while much less so in two Leiomyosarcoma (LMS) cell lines (IC_50_: 346.90 nM for SKUT-1, and 4.17 µM for CP0024). In comparison, other tested compounds either showed poor differential effects between SFT and LMS cell lines (Figs. 1**c**, 1**d**, and 1**e** for Zinc Pyrithione, Neratinib, and TAK-901, respectively) or no clinically meaningful effects in all cell lines (Fig. 1**f** for Pazopanib). As an example (Table 1), the IC_50_ values for Neratinib were 3.61 µM, 0.88 µM, 1.59 µM, and 1.34 µM for INT-SFT, IEC139, SKUT-1, and CP0024 cells, respectively. These results indicated that Mivebresib can efficiently and selectively suppress SFT cells *in vitro*.

**Table 1:** 

Cell Line/IC50 (μM)	Mivebresib	Zinc Pyrithione	Neratinib	TAK-901	Pazopanib
INT-SFT	0.008943	0.3165	3.606	4.269	N/A
IEC139	0.007706	0.03303	0.8757	0.2126	N/A
SK-UT-1	0.3469	0.5049	1.594	0.06597	N/A
CP0024	4.167	0.1693	1.3445	0.3025	N/A

Table 1**. IC50 values for candidate compounds in SFT and LMS cell models.** N/A: not applicable.

### *In vitro* efficacy testing of additional BET inhibitors

**Fig. 2:**
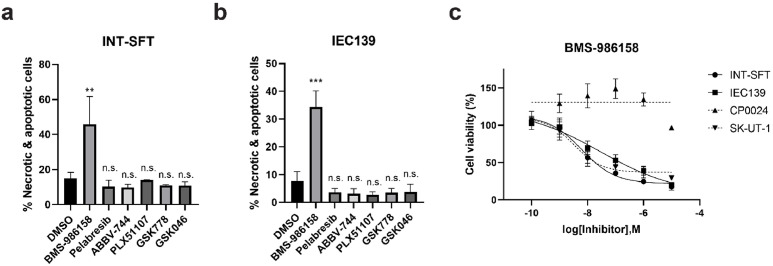
In vitro evaluation of activities and specificities of additional BET inhibitors in SFT cells**. a)***In vitro* evaluation of activities of additional BET inhibitors (BMS-986158, Pelabresib, ABBV-744, PLX51107, GSK778, and GSK046) in INT-SFT cells using Flow cytometry-based apoptosis analysis. **b)***In vitro* evaluation of activities of additional BET inhibitors (BMS-986158, Pelabresib, ABBV-744, PLX51107, GSK778, and GSK046) in IEC139 cells using Flow cytometry-based apoptosis analysis. For both **a)** and **b)**, cells were treated with candidate chemicals (50 nM) for 72 hours, and the drug activities were expressed as the percentage of non-viable cell subpopulation to the total cell population. For statistical analysis, two-tailed t-tests were conducted. ** denotes p<0.01, *** denotes p<0.001, n.s. denotes no significant difference. **c)** Dose-response curves for BMS-986158 at 72 hours in primary SFT cells (INT-SFT and IEC139) and control leiomyosarcoma (CP0024 and SK-UT-1) cells using MTS cell viability assays. Cell viability was normalized to untreated conditions (n=4).

Although Mivebresib, a pan-BETi, showed promising efficacy and specificity in SFT cells (Fig. 1), it is worth noting that in previous phase I clinical trials, some side effects, including thrombocytopenia and anemia, were observed [35,36]. Therefore, we next evaluated six additional BET inhibitors (**Supplementary Table S10**, BMS-986158 [37], Pelabresib [38], ABBV-744 [39], PLX51107 [40], GSK778 [41], and GSK046 [41]) with different targeting selectivity for their *in vitro* efficacy and specificity against SFTs. As shown in Figs. 2**a** and 2**b**, BD1- and BD2-selective BET inhibitors (GSK778 for BD1, ABBV-7444 and GSK046 for BD2), as well as pan-BET inhibitors Pelabresib and PLX51107, did not significantly decrease cell viability in both INT-SFT and IEC139 cells. In contrast, pan-BETi BMS-986158 (50 nM, 72 h treatment) potently induced cell apoptosis and necrosis (45.9 % for INT-SFT, 34.3 % for IEC139) using Annexin V/PI staining-based flow cytometry assay. Similarly, the MTS cell viability assay showed that while BMS-986158 effectively suppressed cell proliferation in INT-SFT and IEC139 cells (IC_50_ values: 6.23 nM for INT-SFT and 28.8 nM for IEC139), it showed no anti-proliferative effects in the LMS cell line CP0024 (Fig. 2**c**). Interestingly, unlike Mivebresib, BMS-986158 also potently suppressed the growth of another LMS cell line SKUT-1 (IC_50_: 3.38 nM). Taken together, these results showed that BMS-986158 demonstrated high efficiency in suppressing the growth of SFT cells *in vitro* but lower SFT specificity compared to Mivebresib.

### BET inhibitors Mivebresib and BMS-986158 induced DNA breaks and G1 cell cycle arrest in SFT cells

**Fig. 3:**
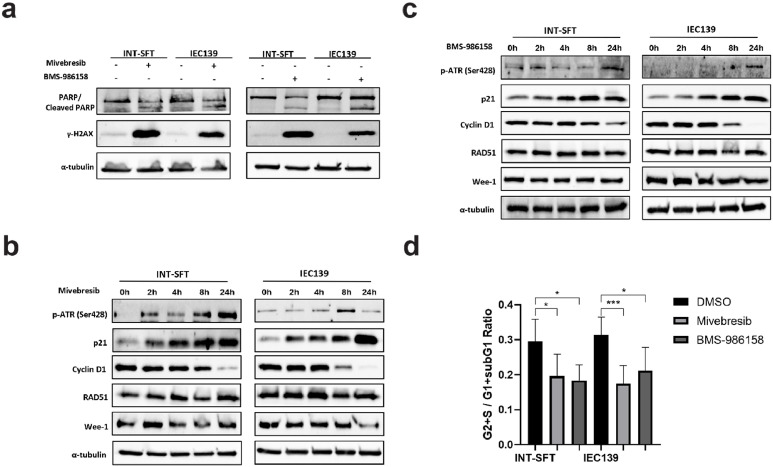
BET inhibitors (BETi) Mivebresib and BMS-986158 exerted anti-proliferative effects in SFTs via cell cycle and DNA damage response pathway**s. a)** Protein levels of apoptosis marker (cleaved PARP-1) and DSBs (double-strand breaks) marker (γ-H2AX) increased in SFT cells after 72-hour treatment with 50 nM of Mivebresib or BMS-986158. **b)** Protein levels of phosphorylated ATR at serine 428 (p-ATR, Ser428), p21, Cyclin D1, RAD51, and Wee-1 in SFT cells (INT-SFT and IEC139) during a 24-hour treatment with 50 nM Mivebresib. The α-tubulin was used as a loading control. **c)** Protein levels of phosphorylated ATR at serine 428 (p-ATR, Ser428), p21, Cyclin D1, RAD51, and Wee-1 in SFT cells (INT-SFT and IEC139) during a 24-hour treatment with 50 nM BMS-986158. The α-tubulin was used as a loading control. d**)** Ratios representing proliferating (G2 and S) vs non-proliferating (sub-G1 and G1) cells upon Mivebresib or BMS-986158 treatment. Bar plots represent mean values with standard deviations. For statistical analysis, two-tailed t-tests were conducted. * denotes p < 0.05; *** denotes p < 0.001.

To uncover the molecular mechanisms of the anti-proliferation effects of Mivebresib and BMS-986158 in SFT cells, we noted that the treatment of these BET inhibitors (50 nM, 72 h) induced the cleavage of PARP-1 (poly(ADP-ribose) polymerase 1, Fig. 3**a**), indicating the activation of apoptosis signaling pathways [42,43]. In addition, the phosphorylation of H2AX at serine 139 (γ-H2AX) [44,45], a recognized marker of double-strand DNA breaks (DSBs) and replication stress, was observed (Fig. 3**a**). More specifically, the treatment of Mivebresib led to a 19.6- and 22.5-fold increase in γ-H2AX levels in INT-SFT and IEC139 cells, respectively, while the treatment of BMS-986158 resulted in a 49.1- and 31.6-fold increase in the same cell lines.

We further analyzed additional protein markers associated with DNA damage response (DDR) pathways or cell cycle regulation (0 to 24 h post-BETi treatment). Interestingly, upregulation of ATR phosphorylation at serine 428 was observed for both BET inhibitors in SFT cell lines (Fig. 3**b** for Mivebresib, Fig. 3**c** for BMS-986158), suggesting that single-strand DNA (SSD) break repair mechanisms were also activated in response to BETi treatments [46]. Specifically, for Mivebresib, ATR phosphorylation peaked at 24 h in INT-SFT cells (a 5.5-fold increase compared to baseline) and at 8 h in IEC139 cells (a 2.9-fold increase compared to baseline). Similarly, for BMS-986158, both cell lines exhibited peak ATR phosphorylation at 24 h (a 2.1-fold increase for INT-SFT and a 3.6-fold increase for IEC139 compared to baseline).

Additionally, elevated levels of p21 [47] and reduced levels of Cyclin D1 [48,49] were observed upon the drug treatments, both of which implied the activation of the DDR pathway and potential G1 cell cycle arrest. Specifically, 24 h after the treatment of Mivebresib, the expression levels of p21 increased by 8.0- and 6.1-fold in INT-SFT and IEC139 cells, respectively. Similarly, for BMS-986158, the expression levels of p21 increased by 2.8-fold in INT-SFT cells and 29.2-fold in IEC139 cells. Next, the expression levels of cyclin D1 decreased by 72.4 % and 98.6 % after 24 h of Mivebresib treatment and by 68.4 % and 90.1 % after BMS-986158 treatment in INT-SFT and IEC139 cells, respectively (Fig. 3**b** and Fig. 3**c**). Reassuringly, these results were consistent with our propidium iodide-based flow cytometry assays. Briefly, 24 h after BET inhibitors treatments, the ratio of proliferating and dividing cells (defined as in S and G2 phases) to non-proliferating cells (defined as in G1 phase and sub-G1 phase for INT-SFT cells, see Materials and methods/Apoptosis and Cell Cycle Analysis by Flow Cytometry) was calculated. As shown in Fig. 3**d**, in both SFT cell lines, treatments with Mivebresib and BMS-986158 significantly decreased such ratios, indicating an accumulation of cells in the G1 phase. As an example, in INT-SFT cells, the ratio for cells under the control treatment (0.29) was significantly higher than those under the treatment of either Mivebresib (0.19, p = 0.047) or BMS-986158 (0.18, p = 0.029). Similarly, in IEC139 cells, the DMSO control group demonstrated a higher ratio (0.31) compared to the Mivebresib (0.17, p < 0.001) and BMS-986158 (0.21, p = 0.026) treated groups.

Lastly, we noted that no significant changes were observed in the expression levels of other DNA repair-related proteins, such as RAD51 and Wee1, suggesting that homologous repair mechanisms (RAD51 as the marker [50,51]) were not activated, and checkpoints beyond the G1 phase of the cell cycle (Wee1 as the marker [52]) remained unaffected (Fig. 3**b** and Fig. 3**c**). Taken together, these findings showed that BET inhibitors Mivebresib and BMS-986158 may suppress the proliferation of SFT cells via the induction of DNA damage and G1 cell cycle arrest.

### Combinatorial effects between BET inhibitors and PARP/ATR inhibitors in SFT cells

Our data showed that BET inhibitors Mivebresib and BMS-986158 induced both double-strand DNA break repair (induction of PARP-1 cleavage) and single-strand DNA break repair (upregulation of ATR phosphorylation) mechanisms in SFT cells. Next, to determine the combinatorial effects between BET inhibitors (Mivebresib: 50 nM, BMS-986158: 50 nM) and DNA damage response pathway inhibitors/DDRi (PARP inhibitor Rucaparib [53]: 10 μM, ATR inhibitor Berzoertib [54]: 1 μM) in SFTs, two assays were utilized: a flow cytometry-based apoptosis assay and Western blot for DDR-related protein markers.

**Fig. 4:**
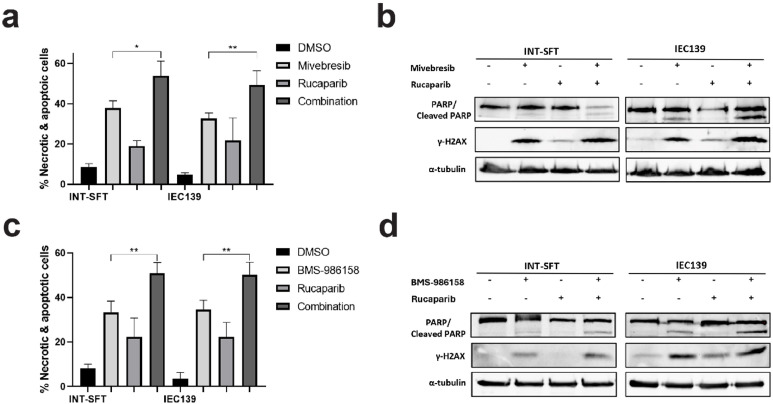
Combinatorial effects between BET inhibitors (Mivebresib and BMS-986158) and PARPi Rucaparib**. a)** Flow cytometry-based apoptosis assays showed that combining Mivebresib and Rucaparib increased apoptotic and necrotic cell populations in INT-SFT and IEC139 cells (n=3). **b)** Western blot assays showed that combining Mivebresib and Rucaparib increased cleaved PARP-1 and γ-H2AX protein levels in INT-SFT and IEC139 cells after 72-hour treatment (n=3). The α-tubulin was used as a loading control. **c)** Flow-cytometry-based apoptosis assays showed that combining BMS-986158 and Rucaparib increased apoptotic and necrotic cell populations in INT-SFT and IEC139 cells (n=3). **d)** Western blot assays showed that combining BMS-986158 and Rucaparib increased cleaved PARP-1 and γ-H2AX protein levels in INT-SFT and IEC139 cells after 72-hour treatment (n=3). The α-tubulin was used as a loading control. For statistical analysis, two-tailed t-tests were conducted. * denotes p < 0.05; ** denotes p < 0.01.

As shown in Fig. 4**a**, compared to Mivebresib alone, the combination of Mivebresib and Rucaparib induced significantly more apoptosis in both INT-SFT and IEC139 cells (1.4- and 1.5-fold increases for INT-SFT and IEC139, respectively). These results were consistent with the finding that the combinatorial drug treatment induced higher expression levels of both cleaved PARP-1 and γ-H2AX compared to Mivebresib alone (Fig. 4**b**, 1.4-fold increase for cleaved PARP-1 in INT-SFT, 2.5-fold increase for γ-H2AX in INT-SFT, 1.6-fold increase for cleaved PARP-1 in IEC139, and 2.3-fold increase for γ-H2AX in IEC139). Similarly, the combination of BMS-986158 and Rucaparib also induced significantly more apoptosis in both INT-SFT and IEC139 cells (Fig. 4**c**, 1.5- and 1.5-fold increases for INT-SFT and IEC139, respectively), as well as higher expression levels of both cleaved PARP-1 and γ-H2AX (Fig. 4**d**, 1.6-fold increase for cleaved PARP-1 in INT-SFT, 1.3-fold increase for γ-H2AX in INT-SFT, 2.1-fold increase for cleaved PARP-1 in IEC139, and 2.9-fold increase for γ-H2AX in IEC139).

**Fig. 5:**
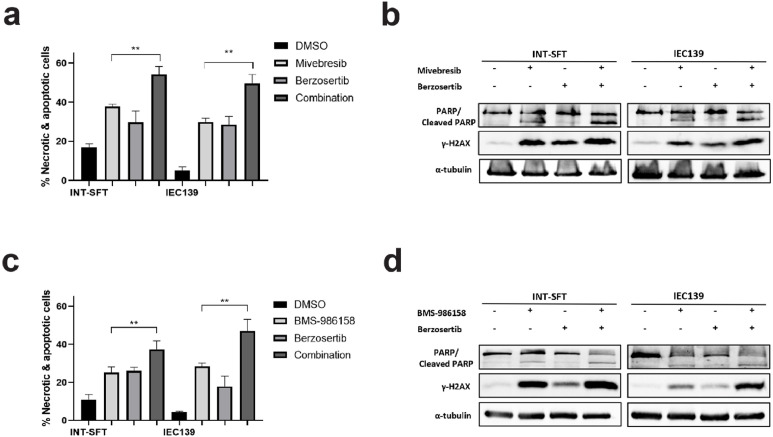
Combinatorial effects between BET inhibitors (Mivebresib and BMS-986158) and ATRi Berzosertib**. a)** Flow-cytometry-based apoptosis assays showed that combining Mivebresib and Berzosertib increased apoptotic and necrotic cell populations in INT-SFT and IEC139 cells (n=3). **b)** Western blot assays showed that combining Mivebresib and Berzosertib increased cleaved PARP-1 and γ-H2AX protein levels in INT-SFT and IEC139 cells after 72-hour treatment (n=3). The α-tubulin was used as a loading control. **c)** Flow-cytometry-based apoptosis assays showed that combining BMS-986158 and Berzosertib increased apoptotic and necrotic cell populations in INT-SFT and IEC139 cells (n=3). **d)** Western blot assays showed that combining BMS-986158 and Berzosertib increased cleaved PARP-1 and γ-H2AX protein levels in INT-SFT and IEC139 cells after 72-hour treatment (n=3). The α-tubulin was used as a loading control. For statistical analysis, two-tailed t-tests were conducted. ** denotes p < 0.01.

Similar results were observed when combining BET inhibitors with the ATR inhibitor Berzoertib. As shown in Fig. 5**a**, the combination of Mivebresib and Berzoertib increased cell apoptosis by 1.4- and 1.6-fold compared to Mivebresib alone for INT-SFT and IEC139 cells, respectively (p-values = 0.003 and 0.002, respectively). In addition, the combinatorial drug treatment induced higher expression levels of both cleaved PARP-1 and γ-H2AX compared to Mivebresib alone (Fig. 5**b**, 3.0-fold increase for cleaved PARP-1 in INT-SFT, 1.6-fold increase for γ-H2AX in INT-SFT, 2.1-fold increase for cleaved PARP-1 in IEC139, and 2.0-fold increase for γ-H2AX in IEC139). Similarly, the combination of BMS-986158 and Berzoertib induced more apoptosis in both INT-SFT and IEC139 cells (Fig. 5c, 1.5- and 1.7-fold increases for INT-SFT and IEC139, respectively), as well as higher expression levels of both cleaved PARP-1 and γ-H2AX (Fig. 5**d**, 1.6-fold increase for cleaved PARP-1 in INT-SFT, 1.6-fold increase for γ-H2AX in INT-SFT, 1.3-fold increase for cleaved PARP-1 in IEC139, and 2.3-fold increase for γ-H2AX in IEC139). Thus, our results showed that the combinational treatment between our candidate BET inhibitors and select PARP/ATR inhibitors exerted higher anti-proliferative effects in SFT cells.

Specifically, in INT-SFT cells, the combination treatment increased cell death by 6.1-fold and 2.0-fold compared to the GSK778 treatment and ABBV-744 treatment, respectively. Similarly, in IEC139 cells, the combination treatment increased the non-viable cell population by 13.7-fold and 6.3-fold, compared to the GSK778 treatment and ABBV-744 treatment, respectively.

Synergistic effects were observed for the combination of BETi (Mivebresib and BMS-986158) with Rucaparib in both primary SFT cell lines using the Loewe model, particularly at concentrations around the IC₅₀ of each drug: 10 nM BETi and 10 µM rucaparib (Supplementary Figure S2). In contrast, no Loewe synergy was detected for combinations of BETi with Berzosertib (Supplementary Figure S3). However, synergy between BETi and Berzosertib became evident when analyzed using the HSA model, again most prominently at IC₅₀ concentrations: 10 nM BETi and 1 µM berzosertib (Supplementary Figure S4). We emphasized that synergy found at the IC_50_ concentrations is consistent with the Chou-Talalay isobologram analysis.

### *In vivo* anti-tumor efficacy testing of Mivebresib against SFTs

**Fig. 6:**
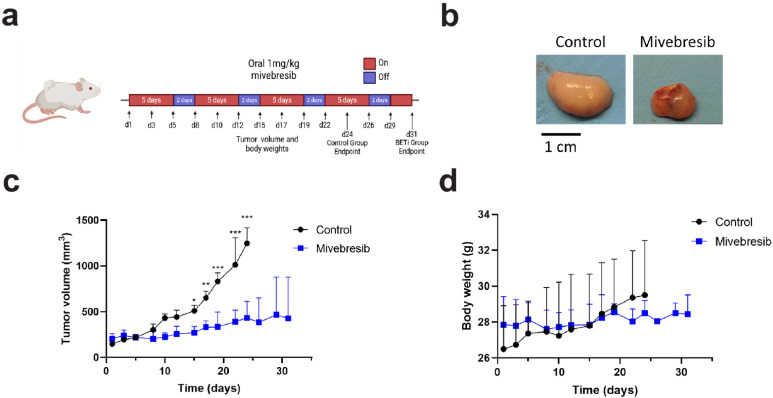
Evaluation of in vivo anti-tumor effects of Mivebresib in IEC139 PDX model**s. a)** Schematic illustration of Mivebresib dosing regimen in IEC139 PDX models. b) Representative images of xenografts harvested at the end of the treatment period. (left) Control, (right) Mivebresib. **c)** Tumor volume progression throughout the treatment period for both Control- and Mivebresib-treated groups (n=3-4 per group). **d)** Body weight measurements throughout the treatment period for Control- and Mivebresib-treated groups (n=3-4 per group). Statistical analysis was conducted using two-way ANOVA with multiple daily comparisons (Sidak). * denotes p < 0.05; ** denotes p < 0.01; *** denotes p < 0.001; non-significant results are not shown.

To evaluate the *in vivo* efficacies of Mivebresib against SFT, an IEC139 patient-derived xenograft (PDX) mouse model was used. Briefly, once IEC139 PDX tumors reach a volume of 150–200 mm³, Mivebresib (dosage: 1 mg/kg body weight, frequency: 5 consecutive days followed by a 2-day break) was administered by oral gavage (Fig. 6**a**). As shown in Figs. 6**b** and 6**c**, treatment with Mivebresib potently reduced tumor volumes compared to the DMSO control. More specifically, 15 days post-treatment, the tumor volumes were 270.0 ± 66.4 mm³ and 509.5 ± 60.9 mm³ for Mivebresib-treated and DMSO-treated groups, respectively. Similarly, 24 days post-treatment, Mivebresib reduced the tumor size by 65.2 % (434.8 ± 178.2 mm³ vs 1247.9 ± 169.5 mm³ for Mivebresib-treated and DMSO-treated groups, respectively). It should also be noted that no significant differences in body weight were observed between Mivebresib- and DMSO-treated groups (Fig. 6**d**), which implied that Mivebresib was generally tolerated *in vivo* at the adopted dosage. In conclusion, our data showed that Mivebresib can exert anti-tumor effects in SFT PDX models.

### Gene expression alterations in SFT cells following BETi treatment

**Fig. 7:**
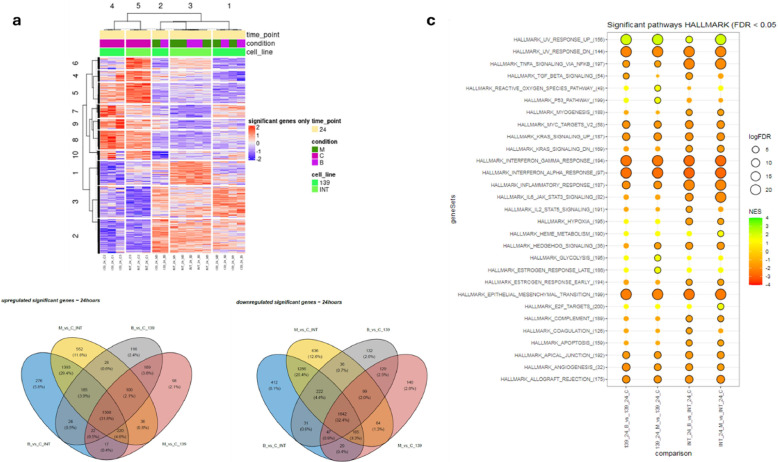
**Transcriptomic response to BET inhibition in SFT cells. a)** Heatmap of differentially expressed genes (DEGs). **b)** Venn diagrams illustrate a substantial overlap of up- and downregulated genes between treatments. **c)** GSEA reveals consistent downregulation of inflammatory, MYC, KRAS, and EMT-related pathways, and upregulation of DNA repair pathways following BET inhibition.

Differentially expressed genes (DEGs) were identified separately for each BETi treatment (Mivebresib and BMS-986158) compared to the corresponding control group. In INT-SFT cells, 4,150 and 3,825 genes were significantly downregulated following Mivebresib and BMS-986158 treatment, respectively, while 4,022 and 3,645 genes were significantly upregulated. Similarly, in IEC139 cells, 2,306 and 2,338 genes were significantly downregulated, and 2,170 and 2,152 genes were significantly upregulated for Mivebresib and BMS-986158, respectively (Supplementary tables S11, S12). Unsupervised hierarchical clustering revealed that while control samples grouped together, treated samples did not cluster by drug type. Instead, samples treated with Mivebresib and BMS-986158 were intermixed, suggesting that both drugs elicited highly similar transcriptional responses in SFT cells (Fig. 7A). Consistent with that hypothesis, 32.4 % of the downregulated genes and 31.8 % of the upregulated genes were shared between the two treatments, indicating a significant overlap in the transcriptional impact of both drugs on SFT cells (Fig. 7B). It should be noted that several known NAB2-STAT6 fusion target genes were found to be downregulated in treated cells compared to controls. For example, IGF2 was downregulated in INT-SFT cells (log₂FC = –1.35, padj < 0.001; log₂FC = –1.25, padj < 0.001 for Mivebresib and BMS-986158, respectively), FGF2 in INT-SFT (log₂FC = –1.67, padj < 0.001; log₂FC = –1.72, padj < 0.001) and IEC139 (log₂FC = –1.00, padj < 0.001; log₂FC = –1.00, padj < 0.001), VEGFA in INT-SFT (log₂FC = –0.47, padj = 0.021; log₂FC = –0.82, padj < 0.001), and CCL26 in INT-SFT (log₂FC = –2.77, padj = 0.001; log₂FC = –3.67, padj < 0.001) and IEC139 (log₂FC = –1.92, padj = 0.046; log₂FC = –2.06, padj = 0.028).

Next, the GSEA analysis identified enrichment in several molecular pathways. Among the enriched Hallmark pathways, the following were consistently downregulated across all four comparisons: UV response, TNFα signaling via NFκB, MYC targets, KRAS signaling (upregulated), interferon-alpha and interferon-gamma signaling, epithelial-mesenchymal transition (EMT), apical junction, angiogenesis, and allograft rejection (Fig. 7C). Notably, several DNA repair pathways were upregulated in the Reactome analysis after BETi treatment, including DNA double-strand break repair, homology-directed repair, and base excision repair (Supplementary table S13).

## Discussion

Our study revealed that two pan-BET inhibitors, Mivebresib and BMS-986158, can potently suppress SFT cell proliferation in both *in vitro* and *in vivo* studies. These results imply that simultaneous inhibition of both bromodomains may be necessary for optimal anti-tumor effects in SFT cells. The combinatorial effects of GSK778 (BD1-selective inhibitor) [55] and ABBV-744 (BD2-selective inhibitor) [56] were analyzed to test this hypothesis. As shown in **Supplementary Figure S5a**, in both INT-SFT and IEC139 cells, the combination treatment induced more pronounced cell apoptosis than the single-agent treatment.

Consistent with cell apoptosis analysis, Western blot showed that the combination treatment induced enhanced PARP-1 cleavage and higher expression levels of γ-H2AX (**Supplementary Figure S5b**), further indicating that dual bromodomain inhibitions were critical for maximizing BETi efficacy in SFT. In line with these results, pan-BET inhibitors demonstrated activity in several other fusion oncoprotein-driven sarcomas. As an example, pan-BET inhibitors have been reported to suppress the transcriptional activity of the EWS::FLI1 transcription factor in both *in vitro* and *in vivo* xenograft experiments in Ewing Sarcoma [21,55,56].

BET inhibitors have been shown to suppress the expression of fusion oncoproteins directly. As an example, Mivebresib treatment was shown to reduce the protein and mRNA levels of EWSR1::ATF1 in a dose-dependent manner in clear cell sarcoma, due to the modulation of BRD4 recruitment at the EWSR1 promoter region [57]. Similarly, as shown in **Supplementary Figure S6a**, following 72 hours of treatment with 50 nM Mivebresib and BMS-986158, the expression of NAB2-STAT6 fusion transcripts in SFT cells was also significantly suppressed. More specifically, in INT-SFT cells, Mivebresib and BMS-986158 treatments reduced NAB2-STAT6 expression by 65.1 % and 51.2 %, respectively. Likewise, in IEC139 cells, Mivebresib and BMS-986158 treatments reduced NAB2-STAT6 expression by 62.6 % and 55.7 %, respectively. Interestingly, other BET inhibitors (Pelabresib, ABBV-744, PLX51107, GSK778, and GSK046), which failed to induce cell apoptosis in SFT cells, did not significantly suppress the NAB2-STAT6 expression. Indeed, as shown in **Supplementary Figure S6b**, a strong positive correlation was observed between the relative expression levels of NAB2-STAT6 and the IC50 values for each BETi (plotted at logarithmic scale, Pearson correlation coefficient = 0.88, p-value < 0.001). These results suggest that BET inhibitors, via downregulating the expression of fusion oncoproteins, may be active across several sarcoma histologies, including SFT [36,58,59].

Next, our results showed that BET inhibitors Mivebresib and BMS-986158 induced DNA breaks and G1 cell cycle arrest in SFT cells (Fig. 3). Importantly, this BETi-induced increase in DNA damage allowed us to test the combinational effects of BET inhibitors with other DNA damage-targeting agents, including PARP and ATR inhibitors (PARP inhibitor Rucaparib, ATR inhibitor Berzosertib, Fig. 4, Fig. 5). We emphasized that, based on the Chou-Talalay analysis (full description in Materials and methods/Chou-Talalay analysis for synergy determination [24]), both BET inhibitors exhibited synergistic effects with either Rucaparib or Berzosertib (except for Mivebresib + Berzosertib in IEC139, which was additive). Specifically, the combination index values are: 0.42 and 0.27 for Mivebresib + Rucaparib; 0.43 and 0.48 for BMS-986158 + Rucaparib; 0.74 and 1.04 for Mivebresib + Berzosertib; and 0.63 and 0.54 for BMS-986158 + Berzosertib in INT-SFT and IEC139 cells, respectively (**Supplementary figures S7-S10**). However, synergy with Berzosertib should be considered only as a potential finding, since it was observed with the HSA model in the combination matrix assays but not with the more appropriate Loewe model. Notably, synergy for BETi + DDRi was observed at IC50 concentrations, which is the concentration range tested by the Chou-Talalay method. It is worth noting that BET inhibitors are implicated in DDR (DNA damage response) pathways in other cancers characterized by elevated genomic instability [[15], [16], [17],[60], [61], [62], [63]]. As an example, Mivebresib was also reported to induce G1 arrest and exhibited dependency on SS18-SSX fusion expression in synovial sarcoma (SS) [64]. More specifically, BET inhibitors were believed to cause DNA damage through multiple mechanisms, including the RNA polymerase II (RNAPII) stalling on the chromatin, and RNA:DNA hybrids (R-loops) accumulation at BET protein binding sites [[65], [66], [67]]. Similarly, the combinational therapy of BET inhibitors and other DNA damage-targeting agents has been applied to other preclinical cancer models, including pancreatic ductal adenocarcinoma [65], ovarian cancer [68], Myc-induced lymphoma cells [69], or melanoma [70]. The cytotoxic effect of this combination appears to be related to several biological processes, including the induction of apoptosis, autophagy, the senescence-associated secretory pathway, and endoplasmic reticulum (ER) stress [70].

Our primary and secondary HTS assays were performed using specific NAB2-STAT6 fusion types (NAB2*_exon6_*::STAT6*_exon17_* for the primary screen and NAB2*_exon5_*::STAT6*_exon16_* for the secondary screen). To further test the robustness of our candidate compounds, after the completion of the primary and secondary HTS assays, we further procured two additional SFT patient-derived primary cell lines (INT-SFT with a fusion type: NAB2*_exon6_*::NAB2*_intron6_*::STAT6*_exon16_*; and IEC139 with a fusion type: NAB2*_exon6_*::STAT6*_exon16_*) and performed in vitro confirmatory efficacy testing (MTS Cell Viability assay) [71]. Additionally, to determine the specificity of our hits against SFTs, but not other soft tissue sarcomas, two Leiomyosarcoma cell lines (SKUT-1 and CP0024) were included.

We are aware that different SFT models, engineered or isolated from patient tissues, were used in our screening, mainly due to the rarity of SFT and difficulty in culturing SFT from biopsies [22]. Nevertheless, we emphasize that primary SFT cell lines (INT-SFT, IEC139) were used to confirm the efficacy of BET inhibitors. Similarly, another limitation of this study was the limited number of preclinical models of SFT, namely for *in vivo* studies. To overcome this limitation, our team is currently working on collecting fresh tissue from patients diagnosed with SFT to attempt to establish further PDX models. We note that, to our knowledge, the IEC139 is the only non-dedifferentiated PDX model available for SFT-related preclinical research, which reinforces the relevance of the results obtained in this unique animal model. Finally, our current study was limited by the lack of preclinical models featuring the NAB2*_exon4_*::STAT6*_exon2_* fusion type, which would have allowed us to test whether the activity of BET inhibitors is consistent across all SFTs, regardless of the fusion gene breakpoints. While the fusion genes do not seem to impact survival [[72], [73], [74]], we cannot rule out that these breakpoints may affect the efficacy of BET inhibitors in SFT.

The transcriptomic analysis of INT-SFT and IEC139 cells following Mivebresib and BMS-986158 treatment revealed extensive transcriptional alterations, with thousands of DEGs identified in both cell lines. The high degree of overlap in DEGs supports the notion that BETi treatments may converge on common molecular targets despite potential differences in their chemical structures. Hierarchical clustering further reinforced this similarity, as treated samples did not segregate by drug but rather intermixed, contrasting with the distinct clustering observed in control samples. GSEA provided additional insights into the pathways affected by BETi. Downregulation of hallmark pathways involved in tumorigenesis, such as MYC targets, KRAS signaling, TNFα/NFκB signaling, and EMT, suggests that BETi may suppress critical oncogenic and inflammatory pathways that drive SFT growth and metastasis. The consistent downregulation of angiogenesis-related genes is particularly relevant given that antiangiogenic therapies, such as pazopanib, have demonstrated the most promising outcomes in prospective clinical studies of SFT [6,7]. This suggests that BETis might offer a complementary or alternative strategy to disrupt tumor vascularization in SFT. UV response pathway could be linked to DNA damage, as UV radiation is a well-known inducer of DNA lesions.

Interestingly, the upregulation of multiple DDR pathways—including DNA double-strand break repair, homology-directed repair, and base excision repair—suggests a compensatory response to increased genomic stress induced by BETi. This activation of repair mechanisms may reflect an adaptive survival strategy by tumor cells to counteract DNA damage accumulation. Such findings align with previous studies reporting that BETis can disrupt chromatin structure and transcriptional regulation, leading to DNA damage [15]. Consistent with this, the observed synergy between BETis and DDR-targeting agents (PARPi and ATRi) in SFT cells supports the rationale for combination therapies. By simultaneously disrupting transcriptional regulation and impairing DNA repair, this strategy could overwhelm tumor cells' capacity to manage genomic instability, leading to enhanced therapeutic efficacy. However, the mechanism by which BETi causes DNA damage in SFT cells is still to be elucidated.

In conclusion, our study established BET inhibitors Mivebresib and BMS-986158 as novel anti-SFT agents. Future work should focus on identifying, through omics studies, the genetic and epigenetic profiles of SFTs in response to BET inhibitors, which may help reveal the underlying mechanisms of BET inhibitors in the context of NAB2-STAT6 fusion protein in SFTs. Next, we will test the combination of Mivebresib or BMS-986158 with DNA damage-targeting agents, especially PARP inhibitors due to the shown synergy, in *in vivo* models. Finally, a clinical trial should be designed to assess the efficacy of BET inhibitors in SFTs in humans and validate the preclinical findings presented in this work.

## Funding

The content is solely the responsibility of the authors and does not necessarily represent the official views of the National Institutes of Health. LB, JMB, HNH, CAM, DSM, and YL acknowledge funding from the US National Institutes of Health (NIH) grant 1R01CA283330. LB acknowledges funding from the Cecil H. and Ida Green Endowment at the University of Texas at Dallas. HNH acknowledges funding from the University of Texas at Dallas Bioengineering Transform Grant and Vice President Accelerator Award. BP acknowledges the S10 grant, 1S10OD026758-01, which funded the use of the Echo 655 acoustic ejection dispenser in this work. He also wishes to acknowledge the S10 grant, 1S10OD018005-01, which funded the IN Cell Analyzer 6000 high-content imaging platform used in the primary screen. DSM is a recipient of a Miguel Servet contract funded by the National Institute of Health Carlos III (ISCIII) (CP24/00131).

## Data availability

The data collected for this article are available in the **Supplementary Materials**.

## Significance

New therapies are clinically needed for patients with Solitary Fibrous Tumors. We demonstrated that BET inhibitors are highly active in the preclinical setting for treating this sarcoma entity.

## CRediT authorship contribution statement

**Jose L. Mondaza-Hernandez:** Writing – original draft, Validation, Methodology, Investigation, Formal analysis, Data curation, Conceptualization. **David S. Moura:** Writing – original draft, Visualization, Validation, Supervision, Resources, Methodology, Investigation, Funding acquisition, Formal analysis, Data curation, Conceptualization. **Yi Li:** Writing – original draft, Visualization, Validation, Supervision, Methodology, Investigation, Funding acquisition, Formal analysis, Data curation, Conceptualization. **Jesus L. Marti:** Writing – review & editing, Methodology, Formal analysis, Data curation. **Paulino Gomez-Puertas:** Writing – review & editing, Validation, Investigation. **John T. Nguyen:** Writing – review & editing, Validation, Investigation. **Shuguang Wei:** Writing – review & editing, Validation, Methodology, Investigation. **Bruce A. Posner:** Writing – review & editing, Validation, Methodology, Investigation. **Clark A. Meyer:** Writing – original draft, Validation, Project administration, Investigation, Funding acquisition. **Leonidas Bleris:** Writing – original draft, Validation, Supervision, Investigation, Funding acquisition, Formal analysis, Data curation, Conceptualization. **Javier Martin-Broto:** Writing – original draft, Validation, Supervision, Methodology, Investigation, Funding acquisition, Formal analysis, Data curation, Conceptualization. **Heather N. Hayenga:** Writing – original draft, Validation, Supervision, Project administration, Methodology, Investigation, Funding acquisition, Conceptualization.

## Declaration of competing interest

David S. Moura has received institutional research grants from PharmaMar and Synox outside the submitted work; travel support from PharmaMar, and personal fees from Tecnopharma, outside the submitted work. Javier Martin-Broto has received honoraria for consulting or advisory board participation and expert testimony from PharmaMar, Bayer, GSK, Deciphera, Boehringer Ingelheim, Cogent Biosciences, Roche, Tecnofarma, and Asofarma; and research funding for clinical studies (institutional) from Deciphera, PharmaMar, Eli Lilly and Company, BMS, Pfizer, Boehringer Ingelheim, Synox, ABBISKO, Biosplice, Lixte, Karyopharm, Rain Therapeutics, INHIBRX, Immunome, Philogen, Cebiotex, PTC Therapeutics, Inc., and SpringWorks Therapeutics. All the other authors do not have competing interests.

## Appendix Supplementary materials

Image, application 1

Image, application 2

Image, application 3

Image, application 4

Image, application 5

Image, application 6
