# Supplementary material for: Identification of BET inhibitors (BETi) against solitary fibrous tumor (SFT) through high-throughput screening (HTS)
Source: Neoplasia. 2025 Oct 29;70:101244. doi: 10.1016/j.neo.2025.101244 (PMC12603759; doi:10.1016/j.neo.2025.101244)
Supplement: Supplementary file 1 [file mmc1.pdf]

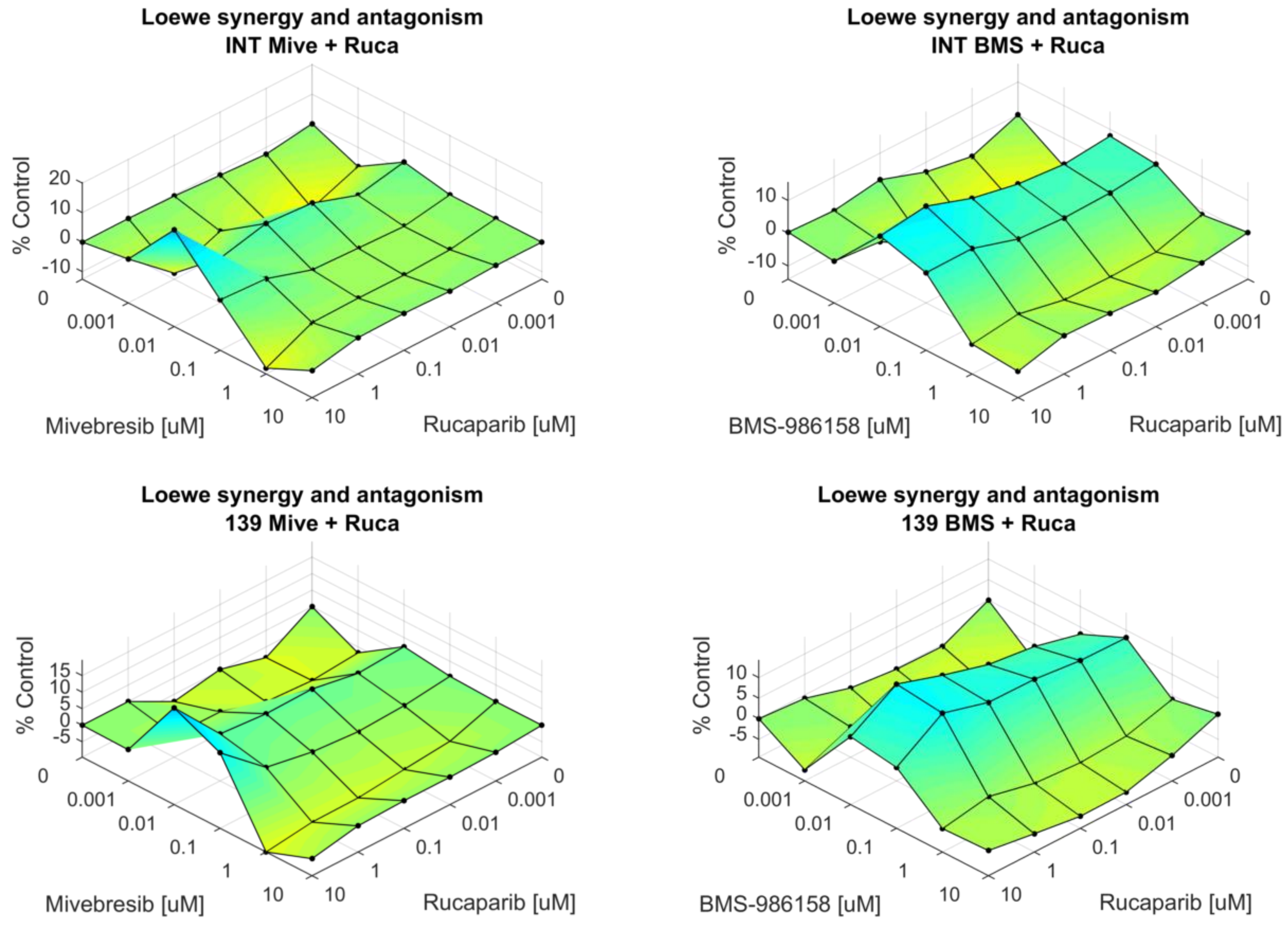

Supplementary Figure 8. Synergy analysis of BET inhibitor (BETi) and Rucaparib combinations using the Loewe model. Top graphs show INT-SFT and bottom graphs IEC139. Synergistic effects are shown in blue, additive effects in green, and antagonistic effects in yellow.

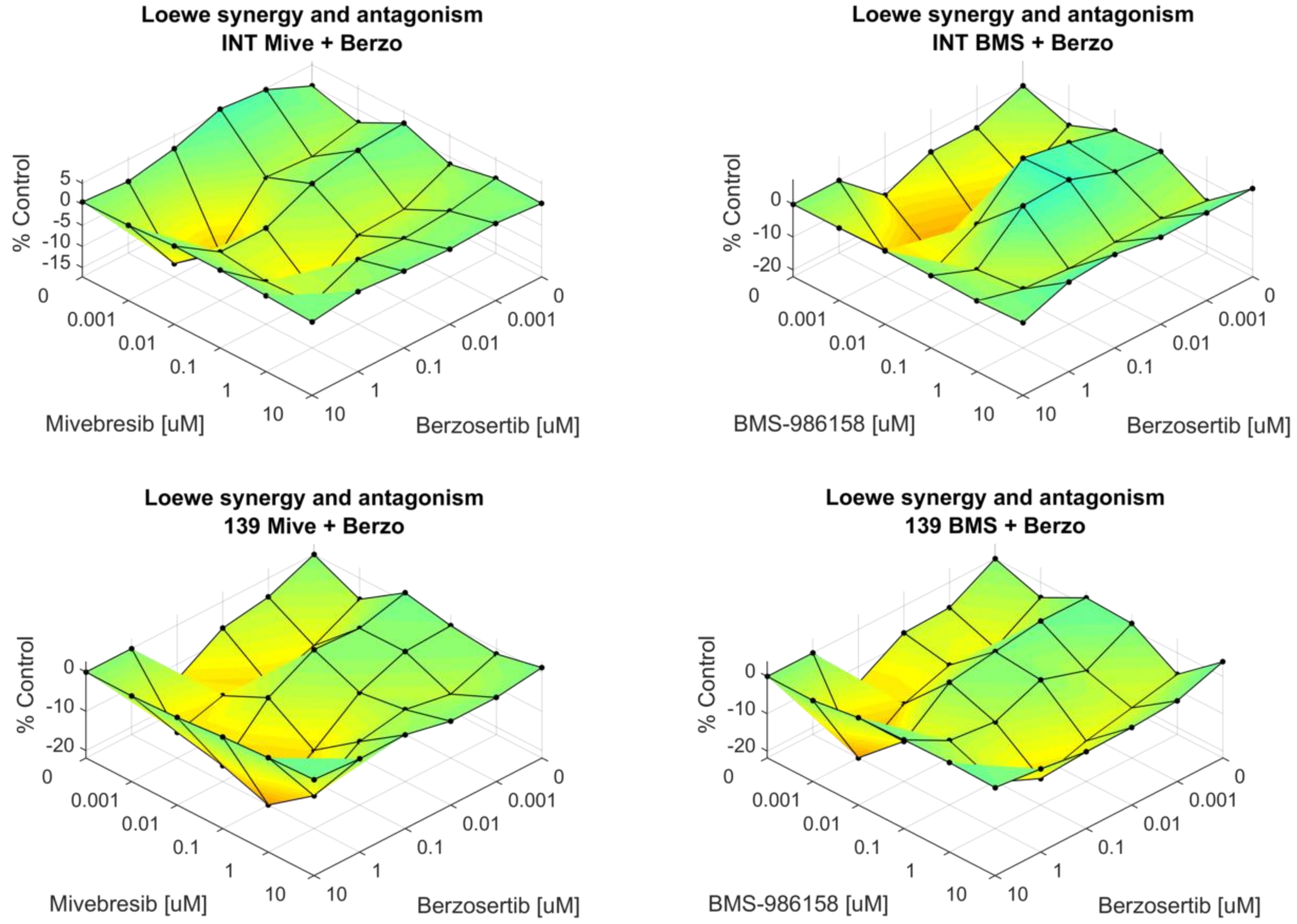

Supplementary Figure 10. Synergy analysis of BET inhibitor (BETi) and Berzosertib combinations using the Loewe model. Top graphs show INT-SFT and bottom graphs IEC139. Synergistic effects are shown in blue, additive effects in green, and antagonistic effects in yellow.

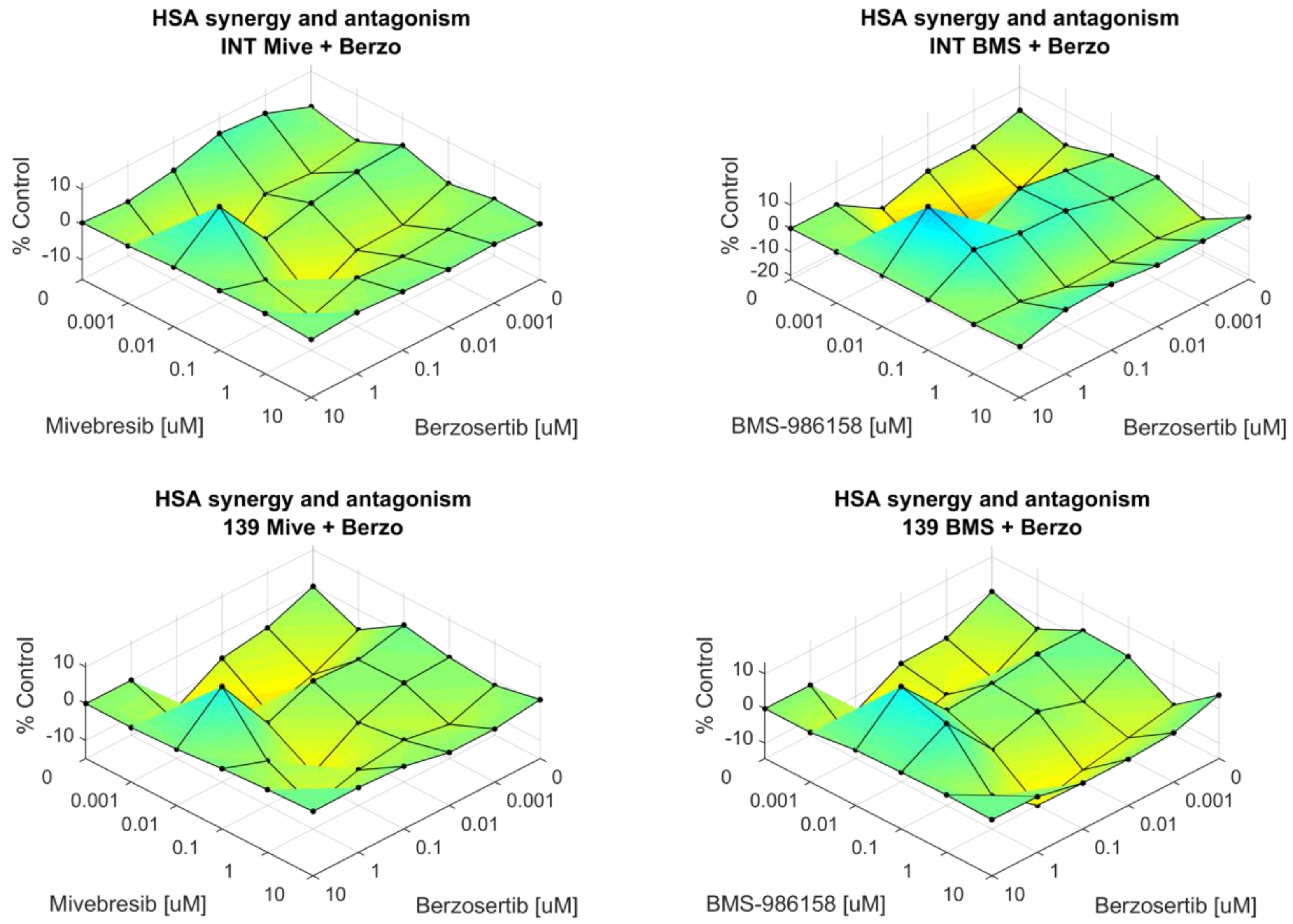

Supplementary Figure 9. Synergy analysis of BET inhibitor (BETi) and Berzosertib combinations using the HSA model. Top graphs show INT-SFT and bottom graphs IEC139. Synergistic effects are shown in blue, additive effects in green, and antagonistic effects in yellow.
