# Supplementary figures and images for: Identification of BET inhibitors (BETi) against solitary fibrous tumor (SFT) through high-throughput screening (HTS)
Source: Neoplasia. 2025 Oct 29;70:101244. doi: 10.1016/j.neo.2025.101244 (PMC12603759; doi:10.1016/j.neo.2025.101244)

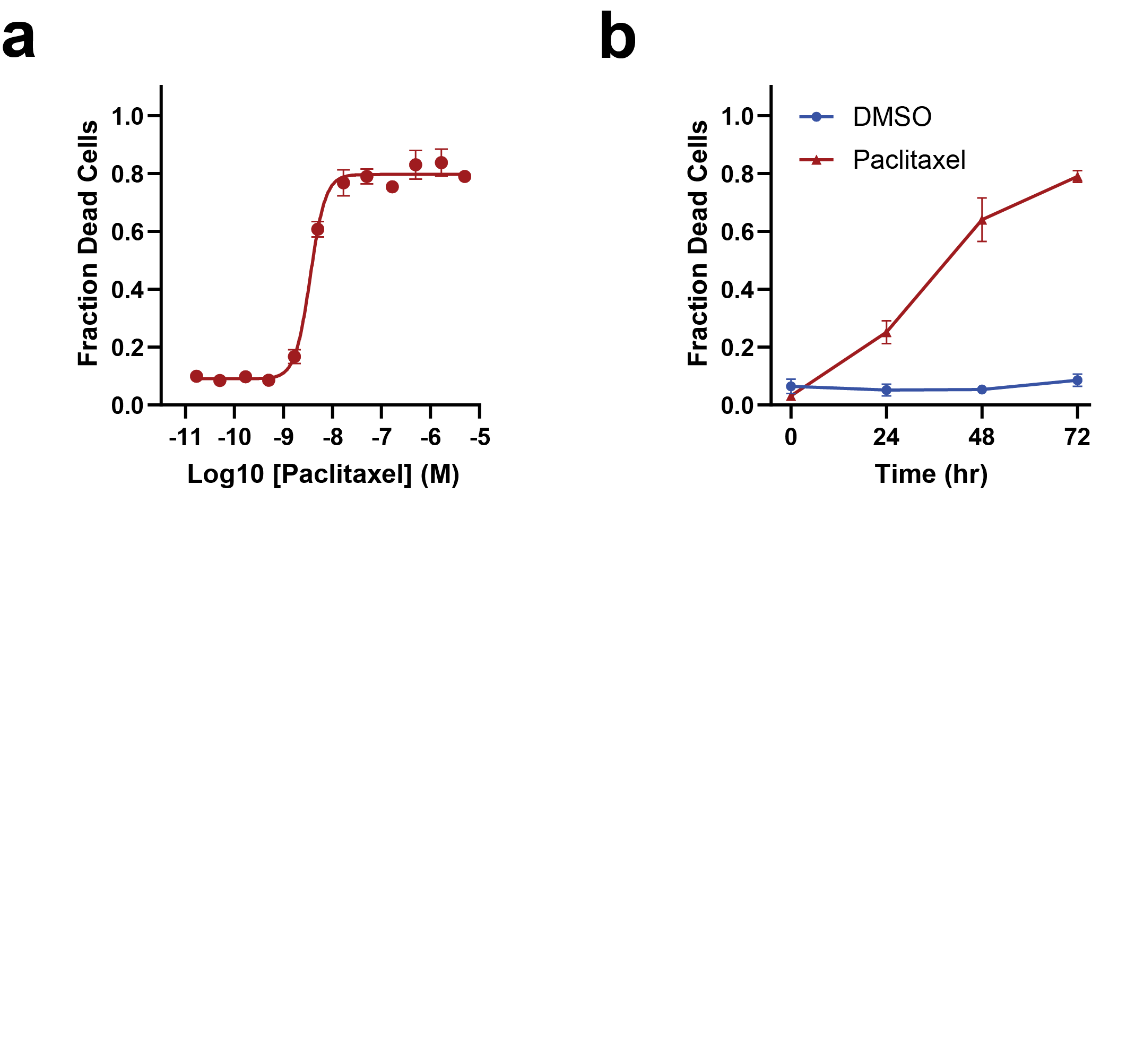

Supplement: Supplementary file 2 [file mmc2.zip › Supplementary_Figure1.png]

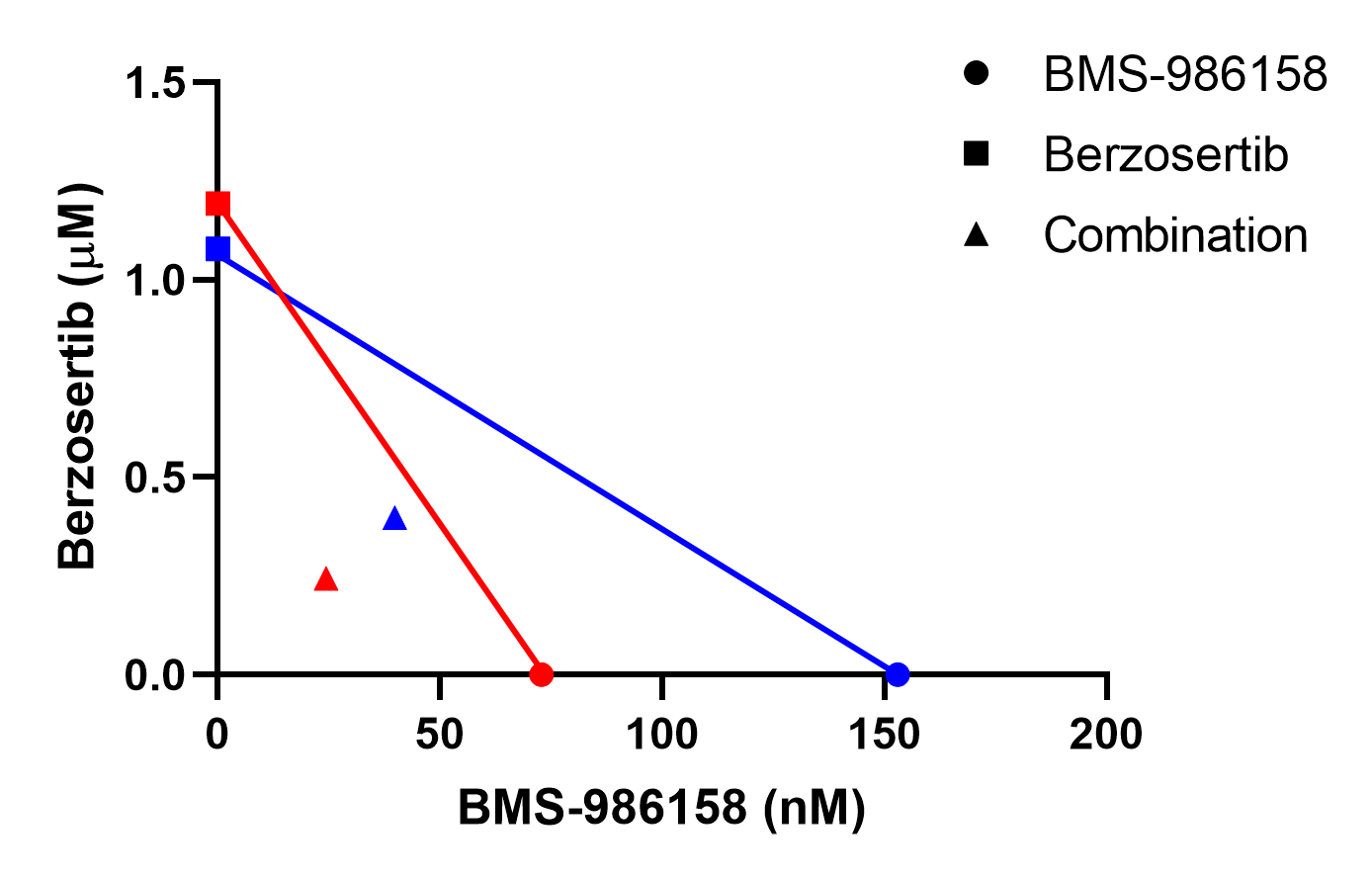

Supplement: Supplementary file 2 [file mmc2.zip › Supplementary_Figure10.png]

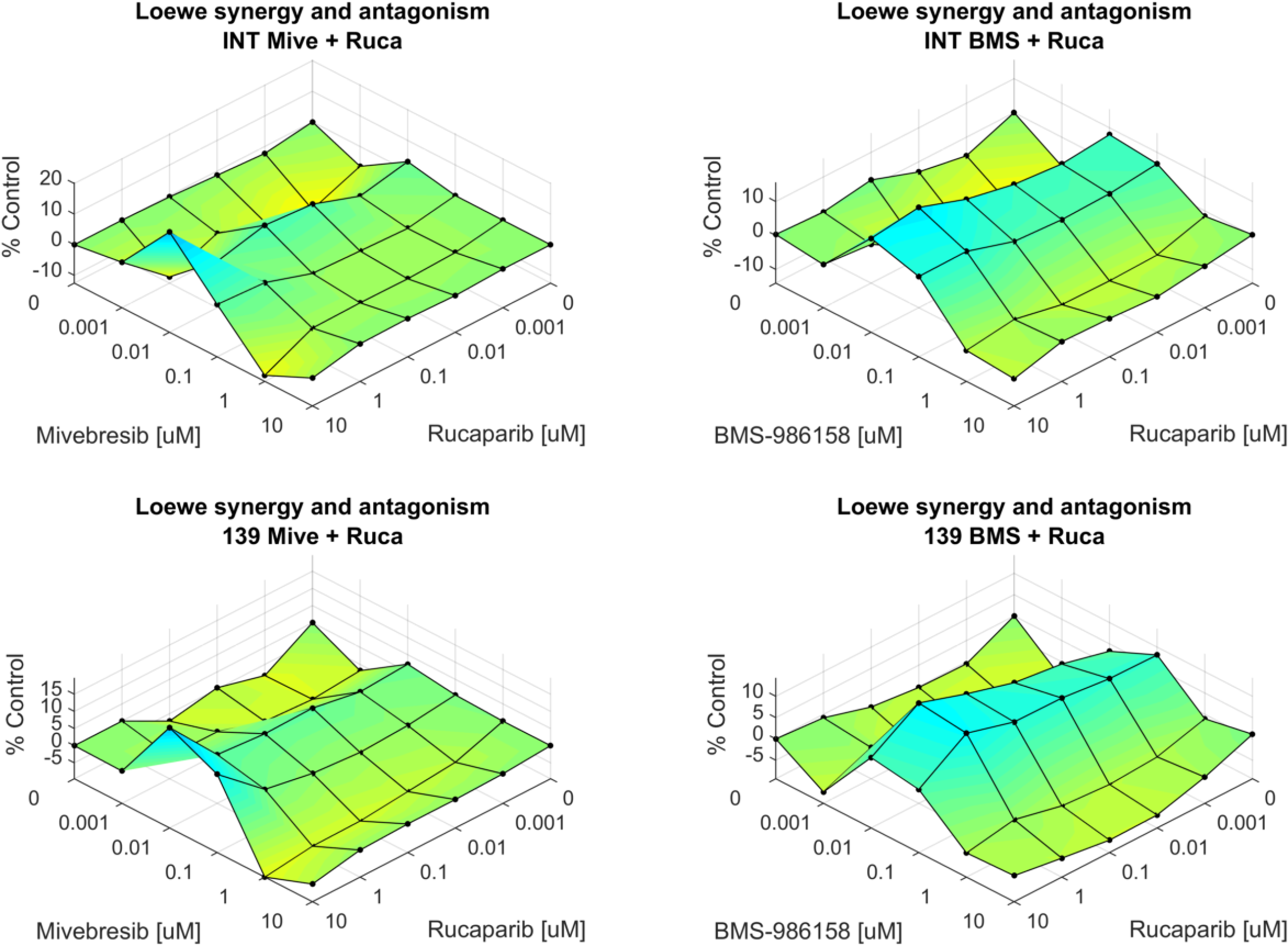

Supplement: Supplementary file 2 [file mmc2.zip › Supplementary_Figure2.png]

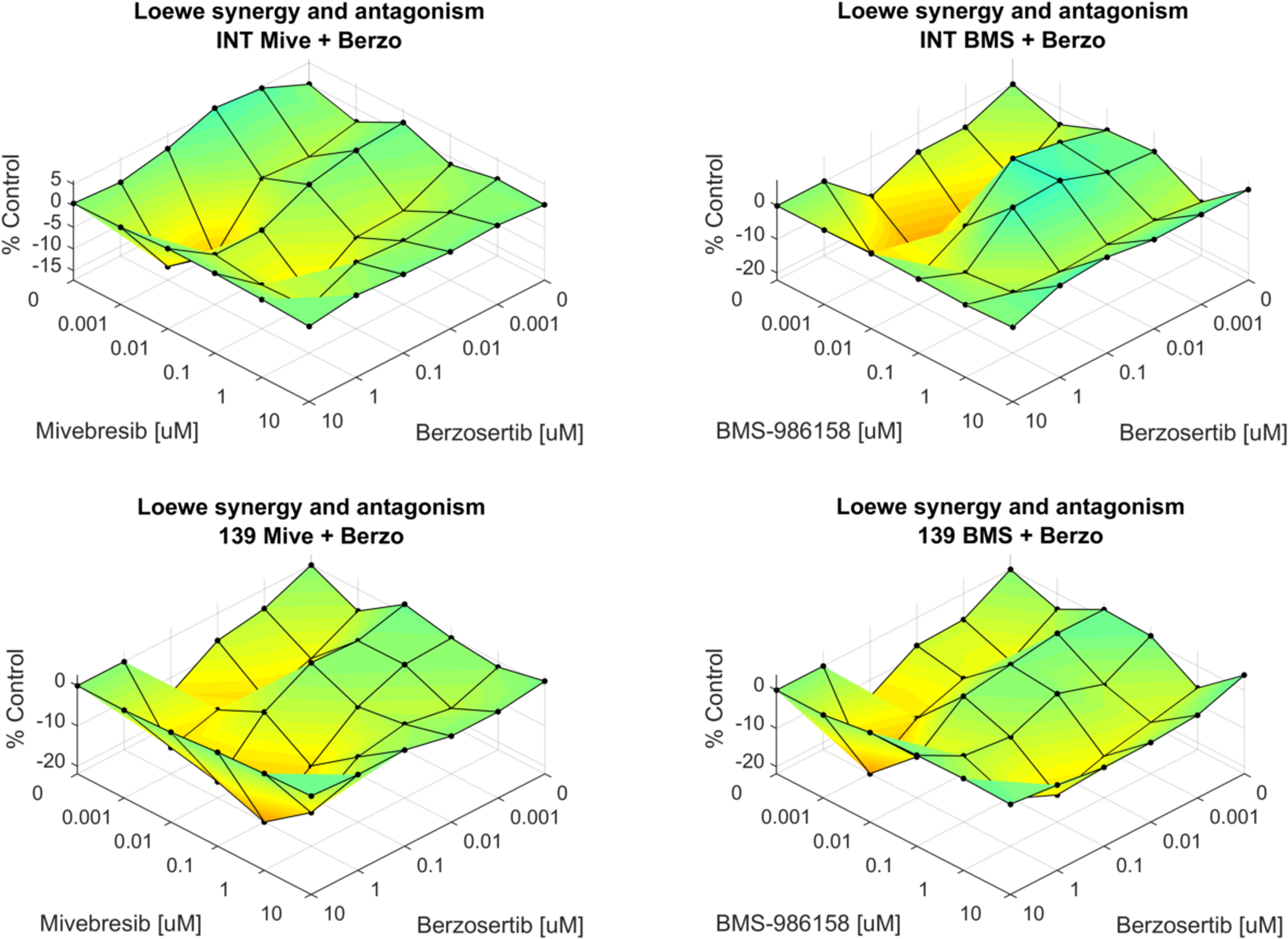

Supplement: Supplementary file 2 [file mmc2.zip › Supplementary_Figure3.png]

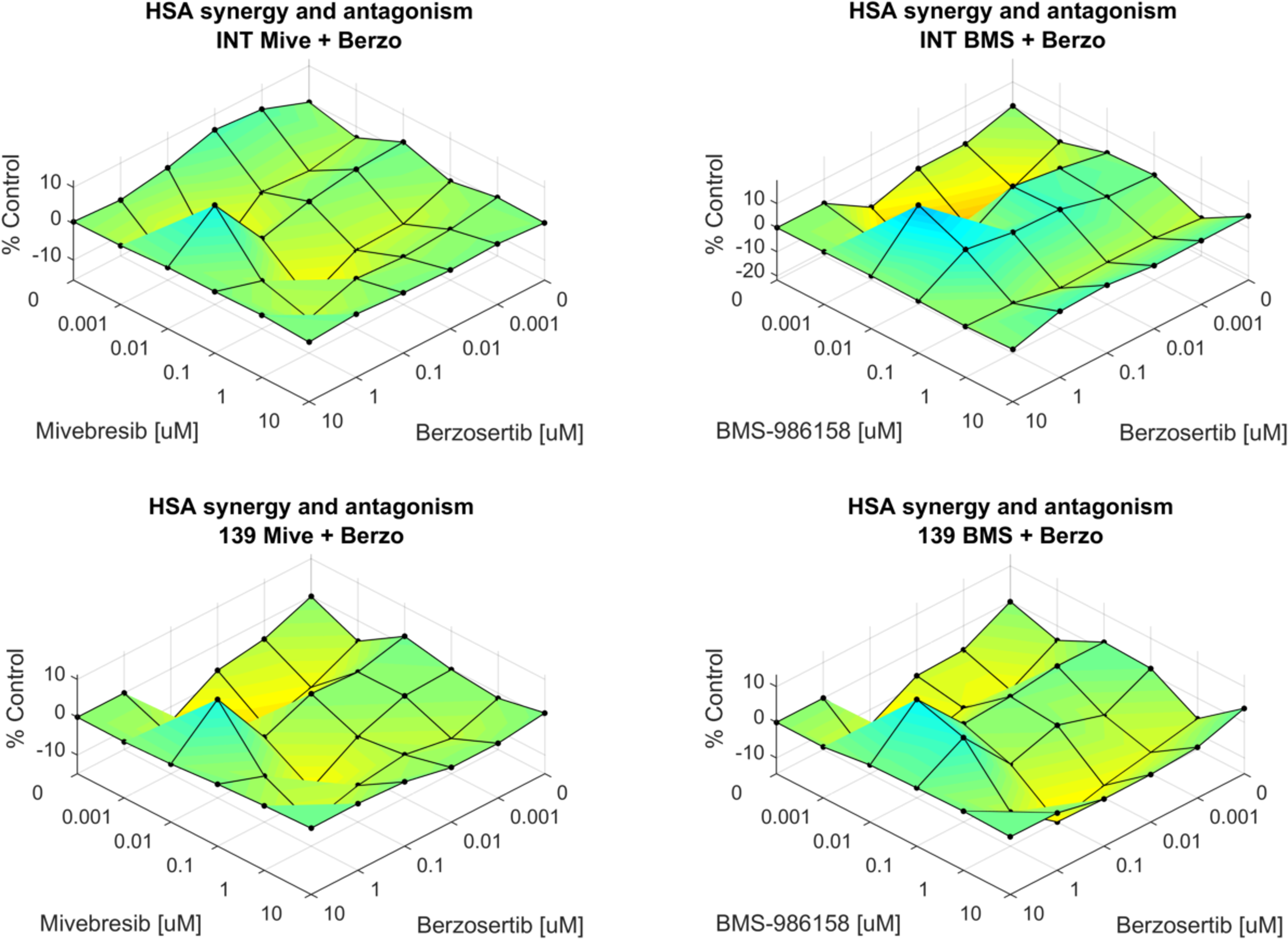

Supplement: Supplementary file 2 [file mmc2.zip › Supplementary_Figure4.png]

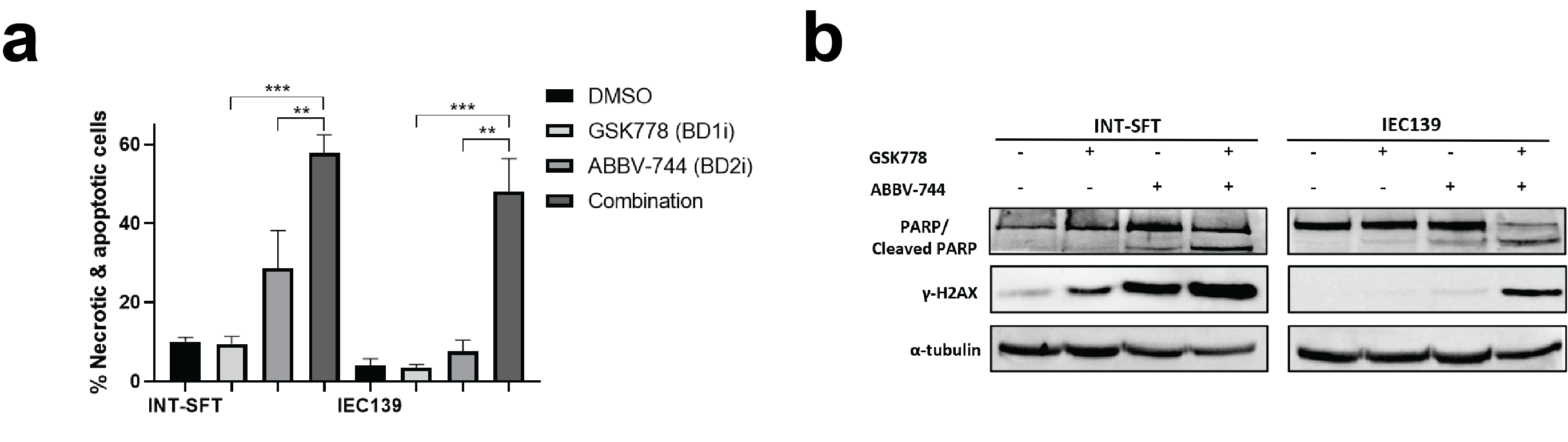

Supplement: Supplementary file 2 [file mmc2.zip › Supplementary_Figure5.png]

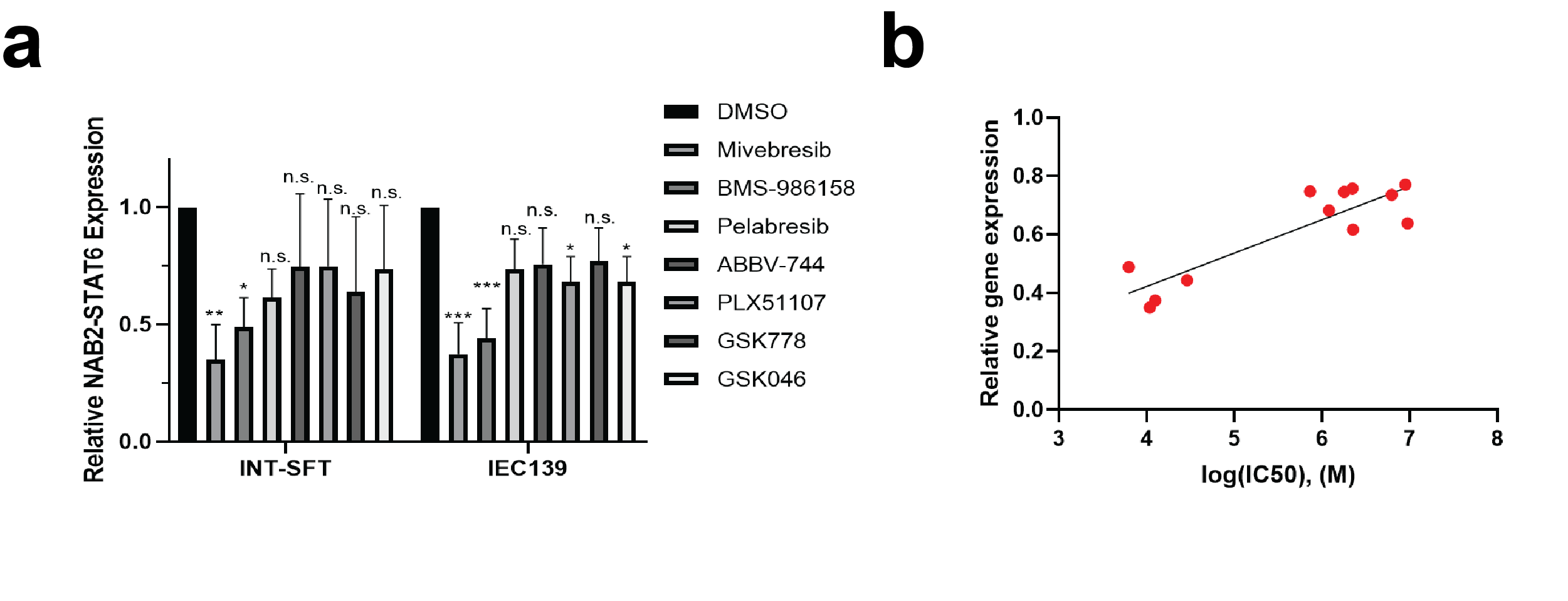

Supplement: Supplementary file 2 [file mmc2.zip › Supplementary_Figure6.png]

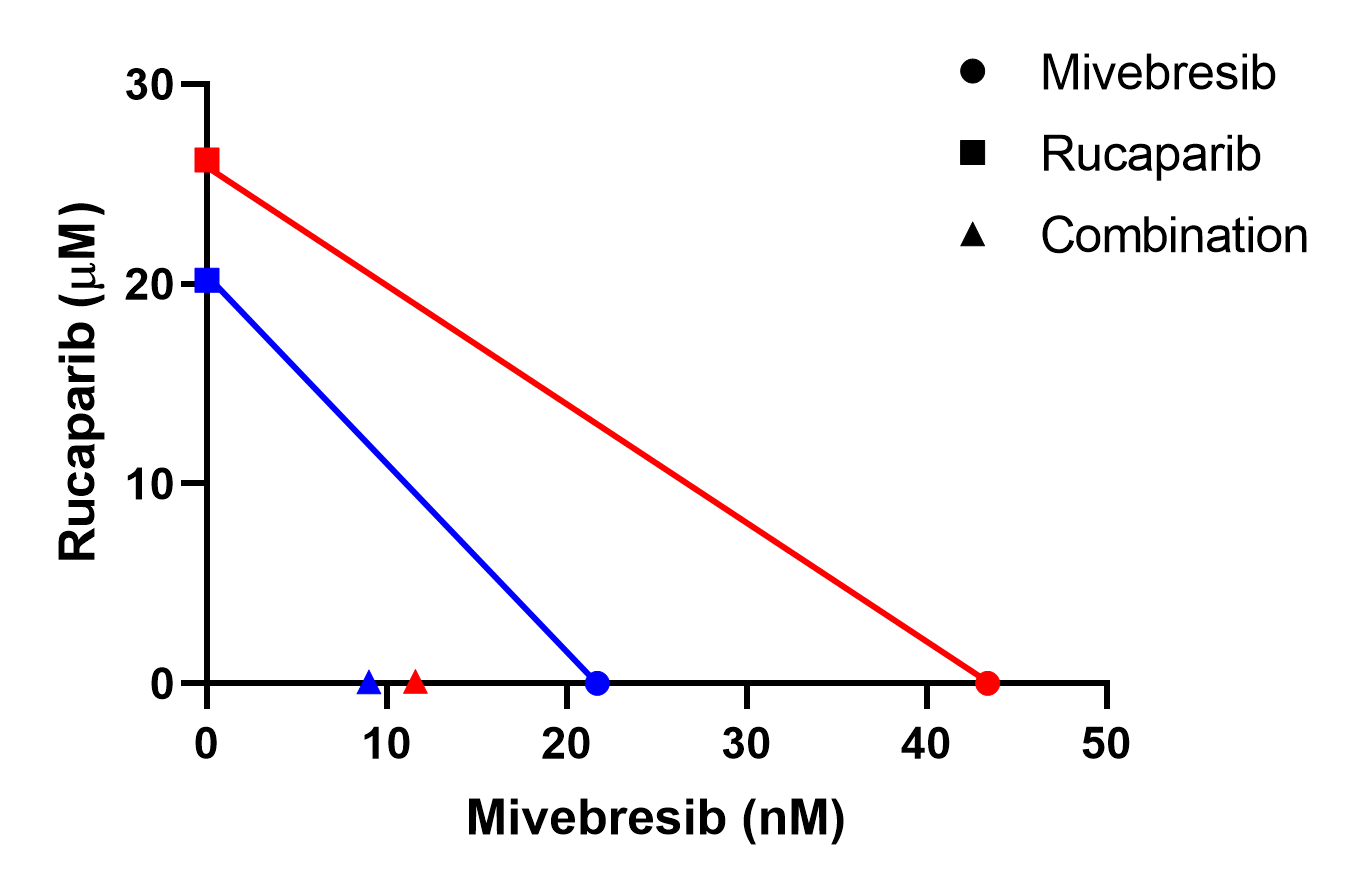

Supplement: Supplementary file 2 [file mmc2.zip › Supplementary_Figure7.png]

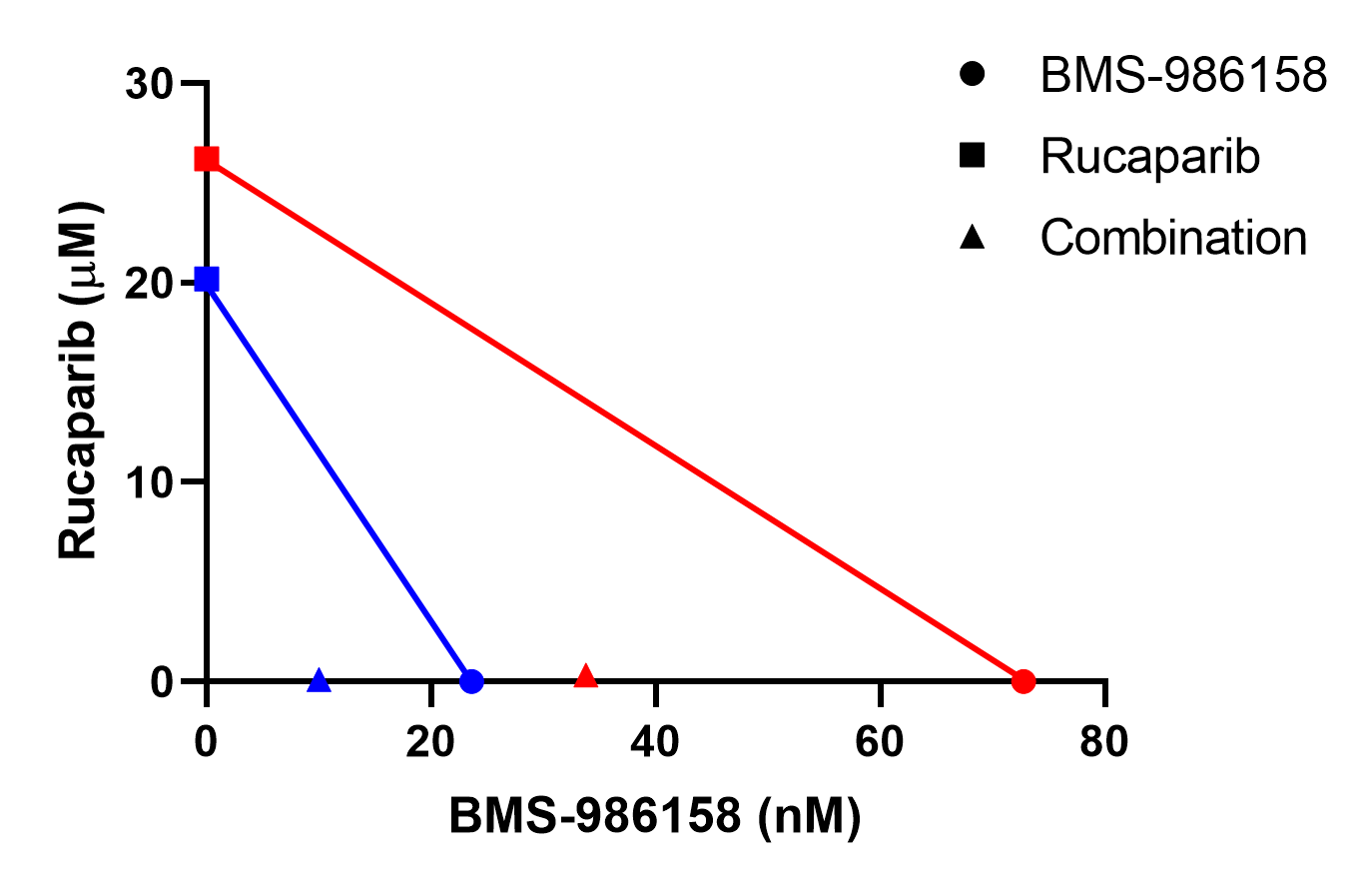

Supplement: Supplementary file 2 [file mmc2.zip › Supplementary_Figure8.png]

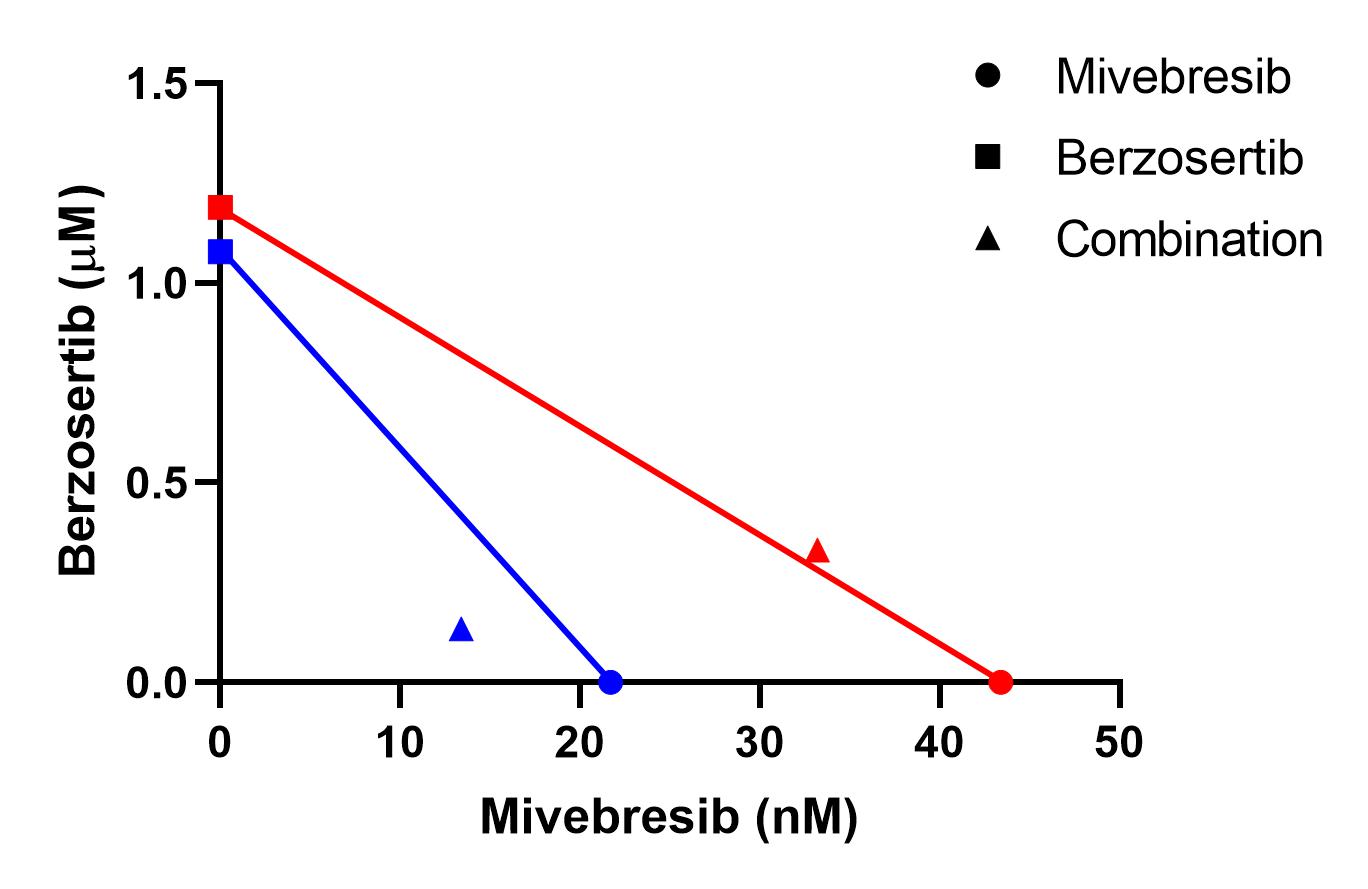

Supplement: Supplementary file 2 [file mmc2.zip › Supplementary_Figure9.jpg]
