# Supplementary material for: Identification of BET inhibitors (BETi) against solitary fibrous tumor (SFT) through high-throughput screening (HTS)
Source: Neoplasia. 2025 Oct 29;70:101244. doi: 10.1016/j.neo.2025.101244 (PMC12603759; doi:10.1016/j.neo.2025.101244)
Supplement: Supplementary file 3 [file mmc3.docx]

**
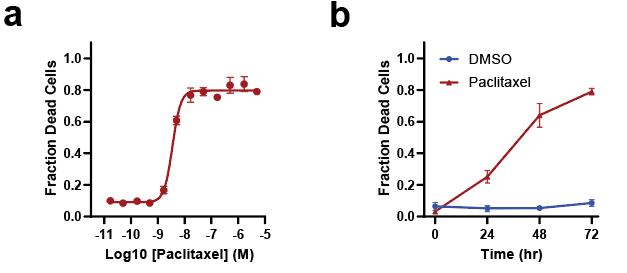
**

**Supplementary Figure 1. High-Throughput Screening Assay for SFT-selective therapeutics.** **(a)** Dose-response curve of NS-poly for Paclitaxel after 72 hours incubation with paclitaxel (IC_50_: 4 nM). **(b)** Time-dependent response NS-poly to 0.5% DMSO (average of 12 wells, blue) and 5 µM Paclitaxel (mean of 3 wells, red). AUC is calculated from these time courses, as detailed in the main text.

**
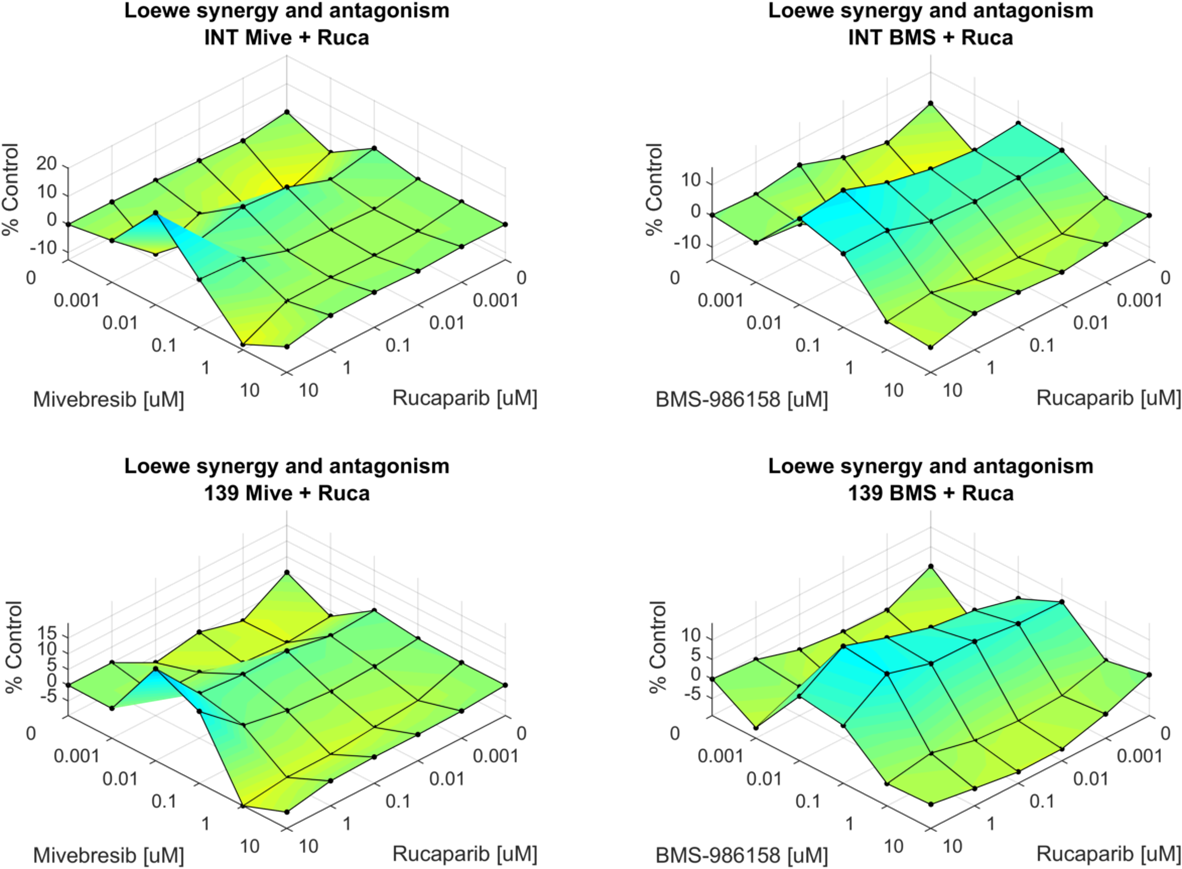
**

**Supplementary Figure 2.** Synergy analysis of BET inhibitor (BETi) and Rucaparib combinations using the Loewe model. Top graphs show INT-SFT and bottom graphs IEC139. Synergistic effects are shown in blue, additive effects in green, and antagonistic effects in yellow.


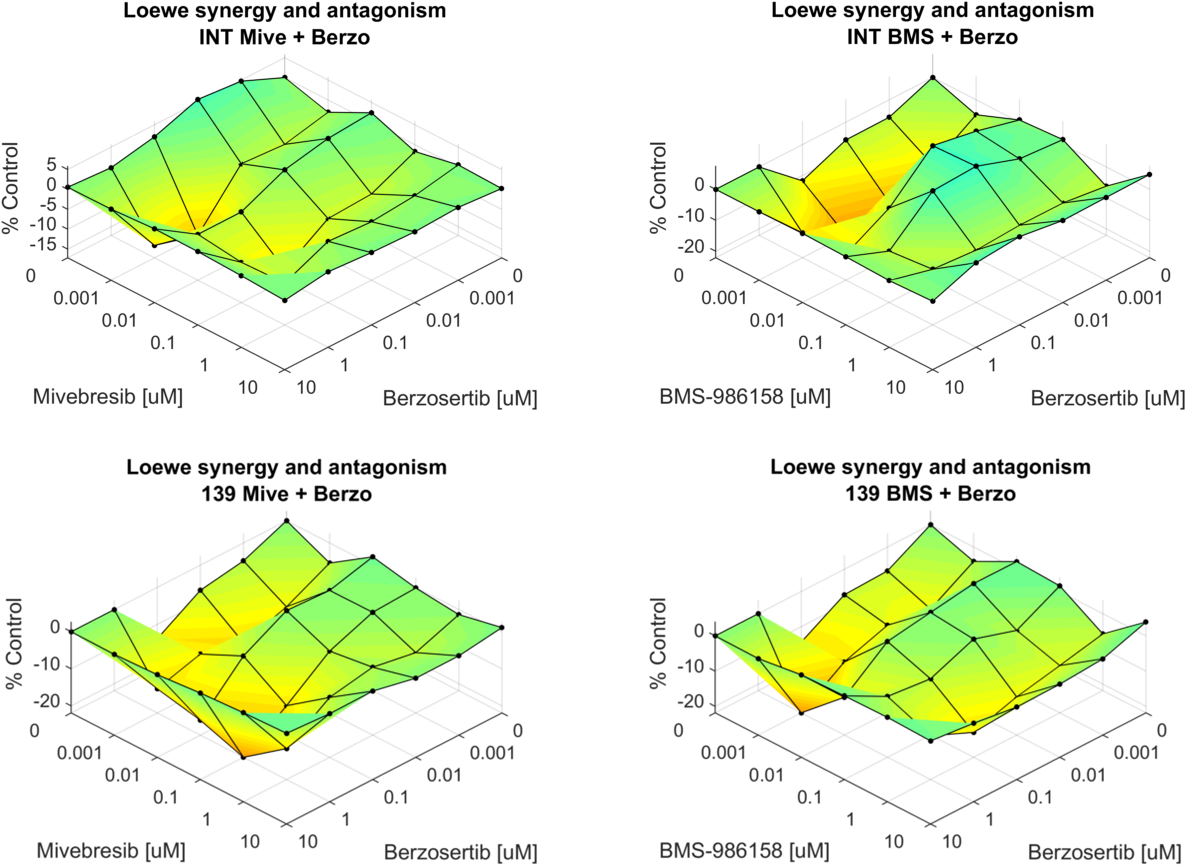


**Supplementary Figure 3**. Synergy analysis of BET inhibitor (BETi) and Berzosertib combinations using the Loewe model. Top graphs show INT-SFT and bottom graphs IEC139. Synergistic effects are shown in blue, additive effects in green, and antagonistic effects in yellow.


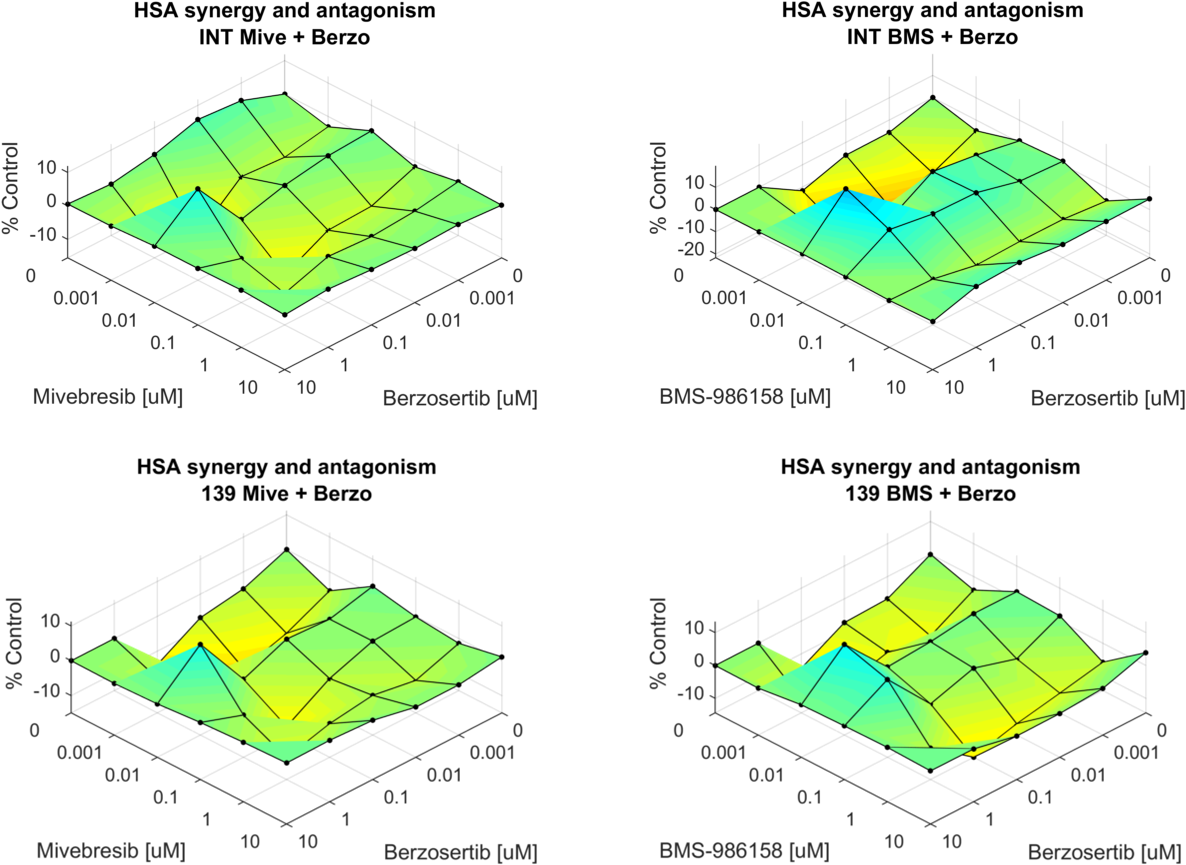


**Supplementary Figure 4**. Synergy analysis of BET inhibitor (BETi) and Berzosertib combinations using the HSA model. Top graphs show INT-SFT and bottom graphs IEC139. Synergistic effects are shown in blue, additive effects in green, and antagonistic effects in yellow.


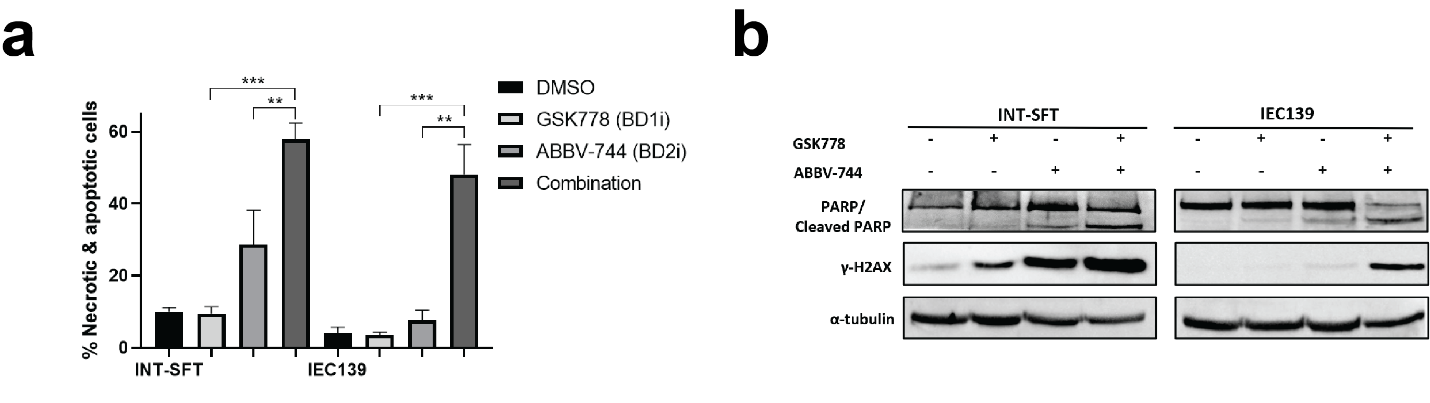


**Supplementary Figure 5. Combinatorial effects between BD1-selective and BD2-selective inhibitors in SFT Cells. a)** Flow cytometry-based apoptosis assays showed that combining GSK778 (1 μM) and ABBV-744 (1 μM) increased apoptotic and necrotic cell populations in INT-SFT and IEC139 cells. **b)** Western blot assays showed that combining GSK778 and ABBV-744 increased cleaved PARP-1 and γ-H2AX protein levels in INT-SFT and IEC139 cells after a 72-hour treatment. For statistical analysis, two-tailed t-tests were conducted. ** denotes p < 0.01; *** denotes p < 0.001.


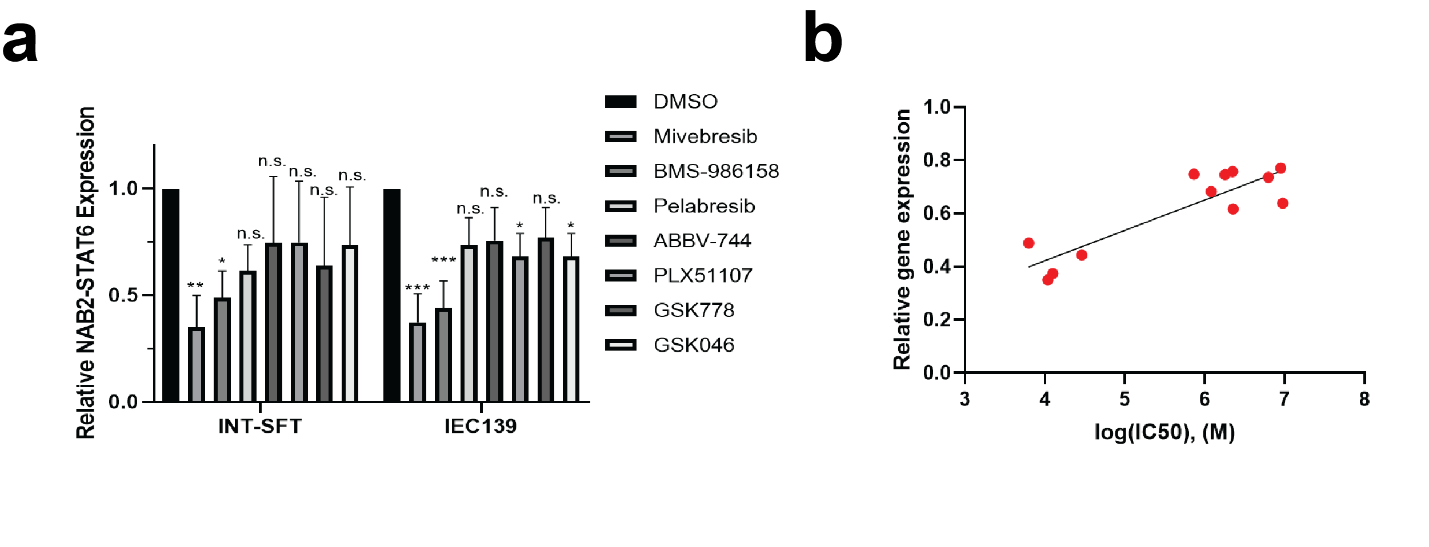


**Supplementary Figure 6.** **BET inhibitors Mivebresib and BMS-986158 downregulated the expression of NAB2-STAT6 in SFT cells.** **a)** Real-time RT-PCR assays showed that the treatments of Mivebresib or BMS-986158 significantly downregulated the expression of NAB2-STAT6 fusion transcripts. (n=4). **b)** Scatter plot showed a positive correlation between NAB2-STAT6 gene expression levels and IC50 values for each BETi treatment (Pearson = 0.88; p-value < 0.001). A one-way ANOVA for each cell line with multiple comparisons to control (Dunett) was performed for statistical analysis. * denotes p < 0.05; ** denotes p< 0.01; *** denotes p < 0.001; n.s., denotes no significant difference.

**Supplementary Figure 7.** **BET inhibitor Mivebresib and PARP inhibitor Rucaparib exhibited synergistic effects in SFT cells (combination index values are 0.42 and 0.27 in INT-SFT and IEC139 cells, respectively). Blue: INT-SFT; Red: IEC139.**


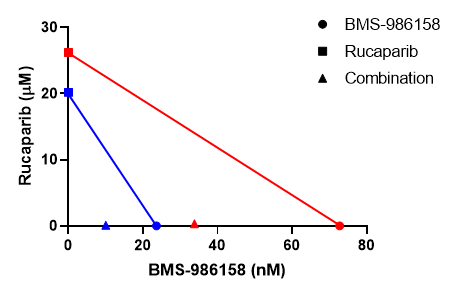


**Supplementary Figure 8.** **BET inhibitor BMS-986158 and PARP inhibitor Rucaparib exhibited synergistic effects in SFT cells (combination index values are 0.43 and 0.48 in INT-SFT and IEC139 cells, respectively). Blue: INT-SFT; Red: IEC139.**

**Supplementary Figure 9.** **BET inhibitor Mivebresib and ATR inhibitor Rucaparib exhibited synergistic effects in SFT cells (combination index values are 0.74 and 1.04 in INT-SFT and IEC139 cells, respectively). Blue: INT-SFT; Red: IEC139.**

**Supplementary Figure 10.** **BET inhibitor BMS-986158 and ATR inhibitor Rucaparib exhibited synergistic effects in SFT cells (combination index values are 0.63 and 0.54 in INT-SFT and IEC139 cells, respectively). Blue: INT-SFT; Red: IEC139.**

**Supplemental materials**

**Supplementary Table 1. Candidate compounds identified from the primary high-throughput screening (HTS) using the final timepoint effects.**

**Supplementary Table 2. Candidate compounds identified from the primary high-throughput screening (HTS) using the AUC effects.**

**Supplementary Table 3. Candidate compounds for the secondary high-throughput screening (HTS).**

**Supplementary Table 4. CTG effects of candidate compounds procured from NIH in the secondary high-throughput screening (HTS).** The candidate compounds are highlighted in blue.

**Supplementary Table 5. CTG effects of candidate compounds procured from Selleck Chemicals in the secondary high-throughput screening (HTS).** The candidate compounds are highlighted in blue.

**Supplementary Table 6. Final Timepoint effects of candidate compounds procured from NIH in the secondary high-throughput screening (HTS).** The candidate compounds are highlighted in blue.

**Supplementary Table 7. Final Timepoint effects of candidate compounds procured from Selleck Chemicals in the secondary high-throughput screening (HTS).** The candidate compounds are highlighted in blue.

**Supplementary Table 8. AUC effects of candidate compounds procured from NIH in secondary high-throughput screening (HTS).** The candidate compounds are highlighted in blue.

**Supplementary Table 9. AUC effects of candidate compounds procured from Selleck Chemicals in the secondary high-throughput screening (HTS).** The candidate compounds are highlighted in blue.

**Supplementary Table 10. IC50 values for additional BET inhibitors in SFT and LMS cell models.** N/A: not applicable.

**Supplementary Table 11. Upregulated and downregulated differentially expressed genes after BETi (Mivebresib or BMS-986158) treatment in INT-SFT cells.**

**Supplementary table 12. Upregulated and downregulated differentially expressed genes after BETi (Mivebresib or BMS-986158) treatment in IEC139 cells.**

**Supplementary Table 13. Upregulated DNA repair pathways by pathway enrichment analysis (Reactome repository) after BETi treatment.**
